# Gene Expression Modifications by Temperature-Toxicants Interactions in *Caenorhabditis elegans*

**DOI:** 10.1371/journal.pone.0024676

**Published:** 2011-09-09

**Authors:** Ana Viñuela, L. Basten Snoek, Joost A. G. Riksen, Jan E. Kammenga

**Affiliations:** Laboratory of Nematology, Wageningen University, Wageningen, The Netherlands; Ecole Normale Supérieure de Lyon, France

## Abstract

Although organophosphorus pesticides (OP) share a common mode of action, there is increased awareness that they elicit a diverse range of gene expression responses. As yet however, there is no clear understanding of these responses and how they interact with ambient environmental conditions. In the present study, we investigated genome-wide gene expression profiles in the nematode *Caenorhabditis elegans* exposed to two OP, chlorpyrifos and diazinon, in single and combined treatments at different temperatures. Our results show that chlorpyrifos and diazinon induced expression of different genes and that temperature affected the response of detoxification genes to the pesticides. The analysis of transcriptional responses to a combination of chlorpyrifos and diazinon shows interactions between toxicants that affect gene expression. Furthermore, our combined analysis of the transcriptional responses to OP at different temperatures suggests that the combination of OP and high temperatures affect detoxification genes and modified the toxic levels of the pesticides.

## Introduction

Organophosphorus pesticides (OP) are widely used to eliminate domestic and agricultural pests. Due to this common use, humans and many other organisms are often exposed to combinations of different pesticides. All OP share a common mode of action, namely they inhibit acetylcholinesterase (AChE), leading to a cholinergic hyper stimulation [1]. However, the effect of OP exposure includes secondary targets that are different among members of the OP group [1]–[4]. In addition, treatments of pesticide combinations induce specific gene transcription responses compared to the single treatments [5], [6]. In other words, toxicants with a similar mode-of-action can induce a different molecular response and their combination may affect the toxic response. Toxicity studies of interactions between chemicals are numerous in literature [7]–[10], but studies of gene transcriptional responses are quite limited. Furthermore, other environmental parameters can also interact with toxicants and modify the toxic effect. For example, increased temperature increases the toxicity of OP like diazinon in zebrafish, or chlorpyrifos in earthworms [11], [12]. But very low temperatures do not show significant interactions with pesticides like abamectin and carbendazim in earthworms [13].

The mode of action of OP is determined by the balance between bioactivation and detoxification [14]. Bioactivation of OP occurs in the initial phase of detoxification, when cytochrome P450 enzymes (CYP) and short chain dehydrogenases (SDR) enzymes transform the pesticides into an oxygenated and highly toxic form called oxon-OP [15]. The process usually follows with the effective detoxification (hydrolysis) of the oxon intermediates by UDP-glucuronosyl transferases (UGT) and glutathione-S-transferases (GST) enzymes to a final inactive compound. Direct dearylation of CPF and DZN to this final compound may also be mediated by CYPs in a direct detoxification reaction [15].

*C. elegans* has approximately 80 CYP genes classified in families and subfamilies of which several have been associated to the metabolism of a range of organic and inorganic chemicals [4], [16]–[20]. Menzel *et al*. [17] were among the first to report a systematic gene expression analysis of *C. elegans* CYP genes in response to xenobiotics. The authors found a concentration-dependent relationship of *C. elegans* CYP35A1, A2, A5, and C1 gene expression in response to organic xenobiotics, including a pesticide, showing that biotransformation pathways of OP are also conserved in worms.

Temperature is generally assumed to be positively correlated with toxic effects. This has been attributed to increased uptake and increased accumulation of the toxicant at higher temperatures [21]. Yet, some studies have found a decreased toxicity at higher temperatures in aquatic organisms [22]. This indicates that the metabolic disturbance of a toxicant depends on the temperature. A reason for that may be the temperature effect itself. Temperature modifies the metabolic rate and therefore can have a strong effect on the whole organism [23]. Transcriptional responses to high temperatures have been characterized in many model organisms such as fruit flies (*Drosophila melanogaster*), springtails (*Folsomia candida*) and *C. elegans*, among others [24]–[26].

Li *et al*. [27] mapped genetic determinants for gene expression at different temperatures and for gene-environment interactions in *C. elegans*. Their results indicated that gene expression regulation differs with temperature, and strongly suggest that the interaction between toxicants and temperature also affected transcriptional responses. To investigate possible transcriptional responses to multiple interacting factors we analyzed genome-wide gene expression profiles using microarrays of the *C. elegans* strain N2 in different environments. We hypothesized that higher temperatures would modify the gene regulatory network in such a way that the regulated genes by toxicants change, and not necessarily their expression levels. Nematodes were treated with two OP, chlorpyrifos (CPF) and diazinon (DZN) in a single and combined (low dose) treatment at 24°C. Then, those gene expression data were combined with previously published expression profiles with identical toxicant treatments at 16°C [28]. Both experiments however were conducted at the same time thus excluding any potential batch effects. Our analysis focused on the identification of responsive genes to the different factors under study: CPF, DZN and temperature. The expression profiles allowed us to identify those genes and biological functions which were affected by interactions between temperature and OP.

## Results

### OP treatments regulated expression of different genes

**Figure 1:**
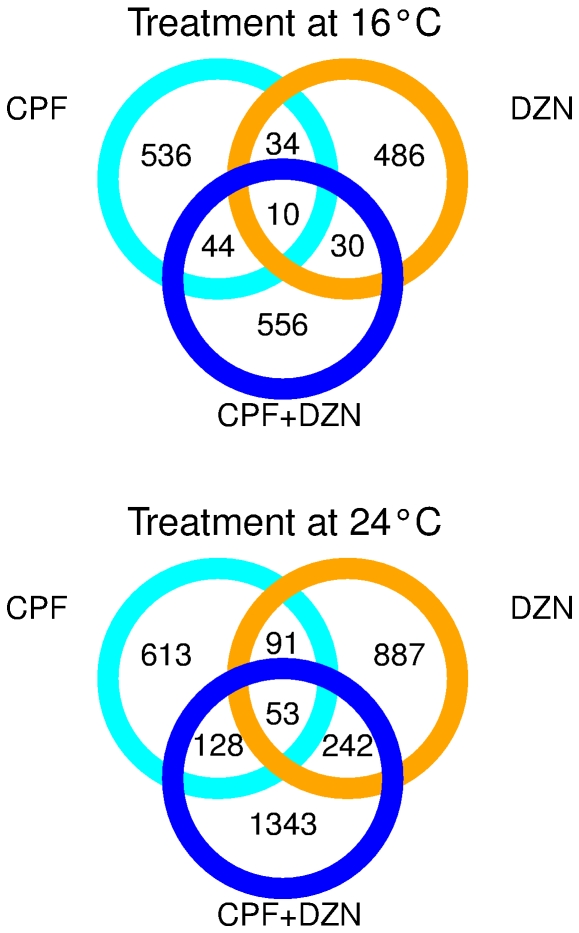
Differentially expressed genes in response to CPF, DZN and combination at 16°C and 24°C. Venn diagram showing significantly regulated genes by CPF (cyan circle), DZN (orange circle), a combination of both (CPF + DZN, blue circle) and their overlap at two temperatures.

First, we investigated the expression profiles from nematodes treated with CPF, DZN and a combination of both (CPF+DZN) at 24°C. Simultaneously, we re-analyzed expression profiles previously published [28] from nematodes treated with identical toxicant concentrations and rearing treatments at 16°C. Figure 1 shows two Venn diagrams with the differentially expressed genes at both temperatures, and the overlap between the different toxicant treatments (Table S1). In general, more genes were significantly regulated at 24°C compared to 16°C in all treatments. Genes significantly regulated by CPF, DZN and CPF+DZN treatments were 4.7% (885), 6.7% (1273) and 9.3% (1766) of the total number of tested genes (18889), respectively. At 16°C, CPF regulated 3.3% of all the genes (624), DZN 3.0% (560), and the combined treatment CPF+DZN 3.4% (640). At both temperatures, the number of genes commonly regulated by the three treatments was small, with 10 and 53 genes at 16°C and 24°C, respectively (Table S2).

**Figure 2 pone-0024676-g002:**
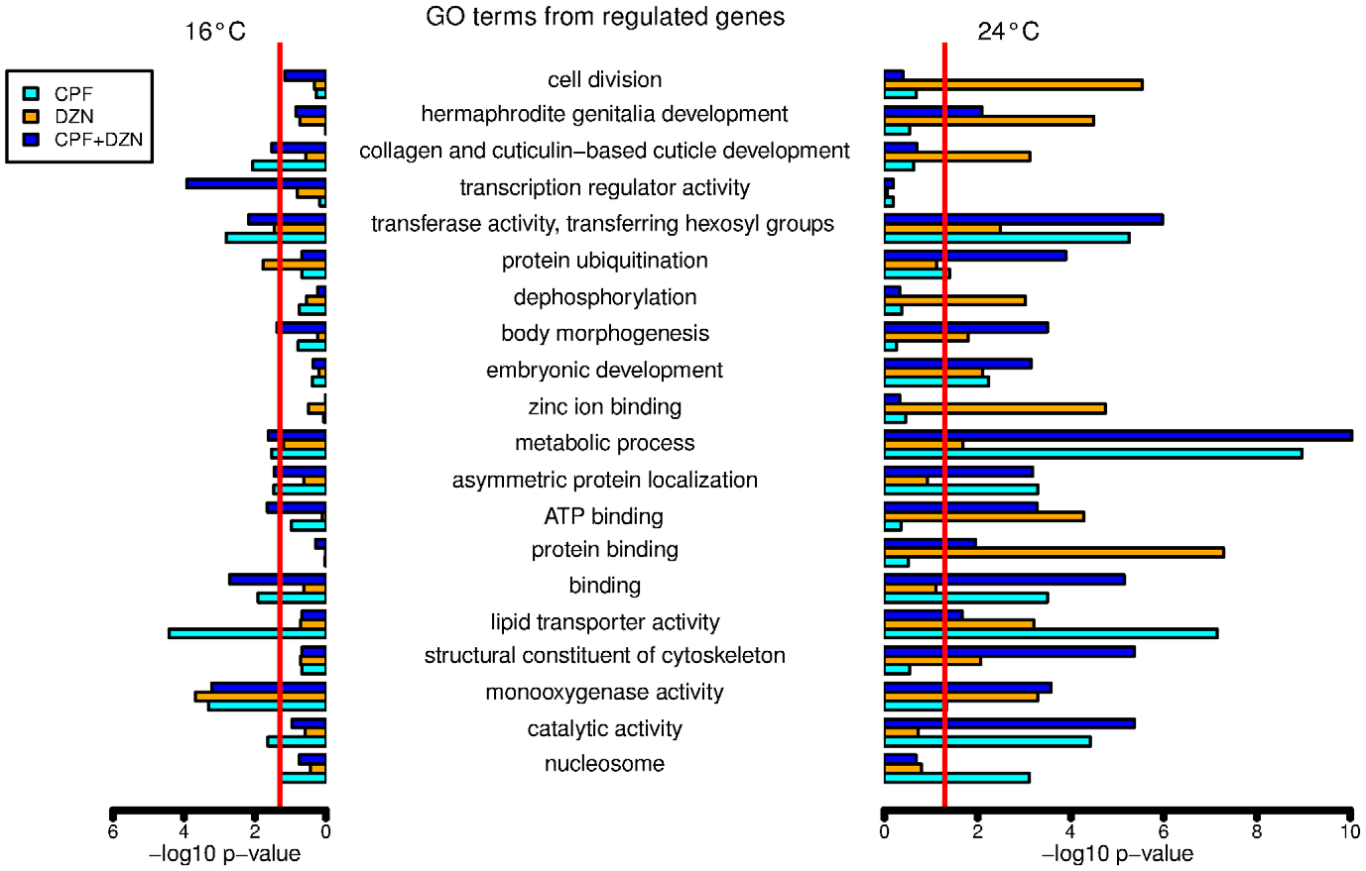
Highly significant Gene Ontology (GO) enriched terms from differentially expressed genes in CPF, DZN and CPF+DZN combination treatments at 16°C and 24°C. As a summary from GO analysis, highly significant (p-value  = 0.001, hypergeometric test) enriched GO terms in at least one treatment were selected and the p-values of those GO terms in all treatments were plotted. The full list of GO terms and the corresponding GO ID numbers can be found in Table S3. Each bar identified a treatment: CPF (cyan), DZN (orange) and a combination of both (blue). Left side bars for 16°C treatments and right side bars for 24°C treatments.

We investigated the biological functions associated to the regulated genes using Gene Ontology (GO) information and predicted protein functional domains information. The full list of significantly enriched GO terms and functional domains (p-value 0.05, hypergeometric test) can be found in Table S3. As a summary, we show in Figure 2 highly significant GO terms (p-value <0.001) in at least one treatment, as well as the significant levels for the other treatments. GO terms related to detoxification of OP (monooxygenase activity) and metabolism in general and lipid transport and metabolism were significantly regulated. Moreover, we observed a different effect of temperature on biological functions affected by toxicant treatments. For example, collagen and cuticule development was an enriched biological function in genes affected by CPF and the combination CPF+DZN at 16°C. At higher temperature this function was enriched with genes significantly affected by DZN treatment, but not by other treatments. Similarly, other biological functions were significantly enriched with genes affected by only one treatment, e.g. dephosphorylation and zinc ion binding by DZN at 24°C; or transcription regulator activity by CPF+DZN at 16°C. The analysis of predicted functional domains from the regulated genes by the different treatments is shown in Table S4 (p-value <0.05). Overrepresented domains in all treatments were detoxification domains cytochrome P450 and UDP-gluconosyltransferase, and domain DUF19 with unknown function. Many other domains were enriched like lipid transport and metabolism related domains (e.g. vitellinogen related domains) and innate immunity (e.g. CUB-like domain). The biological functions associated to commonly regulated genes by all three toxicants treatments at both temperatures were also investigated. The low number of commonly regulated genes at 16°C (10 genes) did not shown any significant enrichment for either GO terms or domains. At 24°C, however, three GO terms were significantly enriched (p-value <0.05), and two domains in the 53 genes investigated (Table 1).

**Table 1:** Significantly enriched GO terms in commonly regulated genes at 24°C.

Total Genes in GO	GO term ID	Significant Genes	P-value	Description
**24 °C**				
578	GO:0008152	9	2.19E-06	metabolic process
658	GO:0005488	9	1.23E-05	binding
541	GO:0003824	8	2.94E-05	catalytic activity
**24 °C**				
**Total Genes in Domain**	**Domain ID**	**Significant Genes**	**P-value**	**Description**
95	IPR002198	4	2.52E-06	Short-chain dehydrogenase/reductase
82	IPR002347	4	1.21E-06	Glucose/ribitol dehydrogenase

Gene Ontology (GO) terms and domains enriched (p-value <0.05, hypergeometric test) in the commonly regulated genes (53) by CPF, DZN and the combination CPF+DZN at 24°C (Figure 1). The first column shows the number of genes within each term. GO terms IDs and Domains ID in each treatment are shown, as well as the description of the terms in the final column. Significant genes refers to the number of significantly regulated genes with the GO term or domain.

### Temperature effect on OP regulated genes

**Figure 3 pone-0024676-g003:**
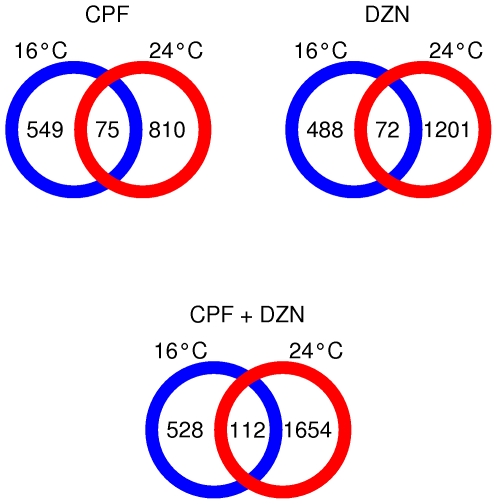
Comparison of differentially expressed genes in response to toxicants at different temperatures. Venn diagram showing significantly regulated genes by CPF, DZN, a combination of both (CPF + DZN) at two temperatures: 16°C (blue circle) and 24°C (red circle).

Toxicant treatments affected a different number of genes at higher and lower temperatures (Figure 1). To better assess the temperature effect, we compared the regulated genes by treatment and between temperatures (Figure 3). In general, we observed little overlap between significantly regulated genes, suggesting a temperature influence on OP responses. The number of commonly regulated genes at different temperatures was 75 for the CPF treatment, 72 for DZN treatment and 112 for CPF+DZN treatment (Table S5). We further investigated the biological functions associated to those genes to gain knowledge on independent transcriptional responses to temperature changes (Table 2). The enriched GO terms included monooxygenase activity, metabolism and binding, all functions associated to detoxification of OP. The functional domain analyses (Table S6) were also related to detoxification domains. In detail, the three treatments had enrichment for cytochrome P450 domains (monooxygenase activity), included in the main detoxification enzymes for OP. Moreover, CPF was also enriched with short chain dehydrogenase/reductase domains; and the combined treatment (CPF+DZN) with UDP-glucoronosyl transferase, which are both detoxification domains as well.

**Table 2:** Biological functions affected by toxicant and independent from temperature effects.

Total Genes in GO	GO terms	Significant Genes	P-value	Description
**CPF**				
578	GO:0008152	12	3.99E-07	metabolic process
85	GO:0004497	5	1.08E-06	monooxygenase activity
658	GO:0005488	11	1.00E-05	binding
541	GO:0003824	9	5.31E-05	catalytic activity
421	GO:0003677	4	2.59E-02	DNA binding
**DZN**				
85	GO:0004497	4	1.80E-05	monooxygenase activity
658	GO:0005488	7	3.48E-03	binding
578	GO:0008152	5	2.27E-02	metabolic process
**CPF + DZN**				
85	GO:0004497	7	3.90E-08	monooxygenase activity
578	GO:0008152	14	1.63E-06	metabolic process
82	GO:0016758	5	9.20E-06	transferase activity, transferring hexosyl groups
98	GO:0016791	4	2.93E-04	phosphatase activity
658	GO:0005488	11	5.30E-04	binding
541	GO:0003824	6	4.20E-02	catalytic activity

Gene Ontology (GO) analysis of the common genes regulated by each treatment at different temperatures (Figure 3). Total Genes in GO are the total number of genes belonging to each GO term. GO terms are the GO identification numbers. Significant genes refer to number of significantly regulated genes in each treatment that belong to a GO term. The description of the GO terms are in the final column.

### Detoxification gene responses to OP treatments and temperature changes

**Figure 4:**
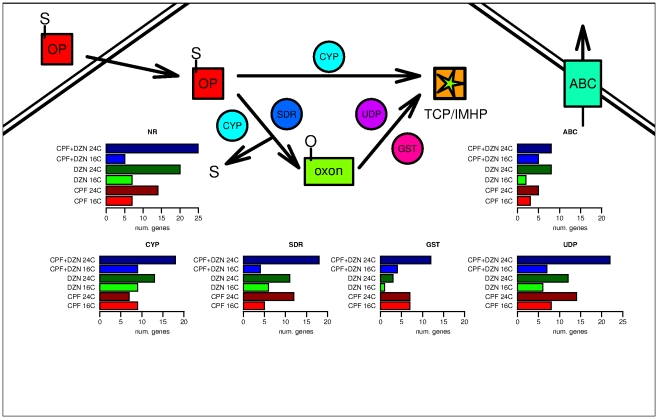
Effects of organophosphate pesticides (OPs) on detoxification genes. Transport of OPs inside the cell activates transcription of nuclear receptor (NR) genes and regulates expression of genes involved in detoxification. An initial phase of CPF and DZN detoxification starts with cytochrome P450 enzymes (CYPs) and short chain dehydrogenases (SDR) enzymes transforming the toxicants into an oxygenated form called oxon-OP (oxon). This highly toxic intermediate metabolite is effectively detoxified by hydrolysis mediated by UDP-glucuronosyl transferases (UGT) and glutathione-S-transferases (GST) enzymes. The final inactive compound of CPF and DZN are 3,5,6-trichloro-2-pyridinol (TCP) and 2-isopropyl-4-methyl-6-hydroxypyrimidine (IMHP), respectively. ABC-transporters (ABC) transport metabolites during the detoxification [28], [29]. The graphs show the number of detoxification genes affected by each toxicant at both temperatures.

Transcriptional regulation of detoxification genes is required in response to all OP exposures in single and combined treatments [4], [28]. To better understand the differences in regulated genes between toxicants we focused on detoxification genes. We included for this analysis the four main detoxification enzymes: cytochrome P450 enzymes (CYP), short chain dehydrogenases (SDR), UDP-glucuronosyl transferases (UGT) and glutathione-S-transferases (GST). But also ATP-binding cassette transporters (ABC) and nuclear receptors (NR), since they are known to be involved in transport of detoxification products and activation of detoxification genes respectively [29]. Figure 4 shows a simplified diagram of the detoxification of OP (CPF and DZN) to their final non-toxic forms and the enzymes involved. We also show the number of genes regulated by each toxicant treatment at both temperatures. At 24°C more detoxification related genes (168 unique genes in all treatments) were significantly regulated than at 16°C (85 unique genes). Only the CPF treatment changed this general trend for CYP genes. Interestingly, at lower temperatures the combination treatment did not differ much from the single treatments in the number of regulated detoxification genes.

The domain analysis (Table S6) showed enrichment for CYP enzymes in all toxicant treatments suggesting that for some CYP the toxicant was a more relevant environmental factor to explain their transcriptional variation than the temperature. Still, and as we showed in Figure 4, more CYP were regulated at higher temperatures indicating that some CYP genes had temperature dependent expression (Table 7A). However, the percentages of detoxification genes from the total number of affected genes by treatment were similar between temperatures. CPF affected 6.2% of detoxification genes at 16°C and 6.6% at 24°C; DZN 5.5% and 5.2%, respectively, and CPF+DZN affected 5.3% and 5.8%. More relevant, however, was the proportion of the different detoxification genes per treatment and temperature (Figure S1). For example, from the total number of detoxification genes (66 and 119 at 16°C and 24°C, respectively) more CYP genes were significantly affected at 16°C and more NR genes were affected at 24°C, suggesting the activation of other pathways at higher temperature. Similarly, SDR domain was enriched for CPF common genes but twice as many genes with SDR domains were regulated at 24°C than at 16°C. Stronger examples were found for genes with UDP domains. The combined treatments have the UDP domain enriched in their commonly regulated genes; however, a much larger number of genes with these domains were regulated at higher temperature.

### Temperature-toxicants interactions modify gene expression

**Figure 5:**
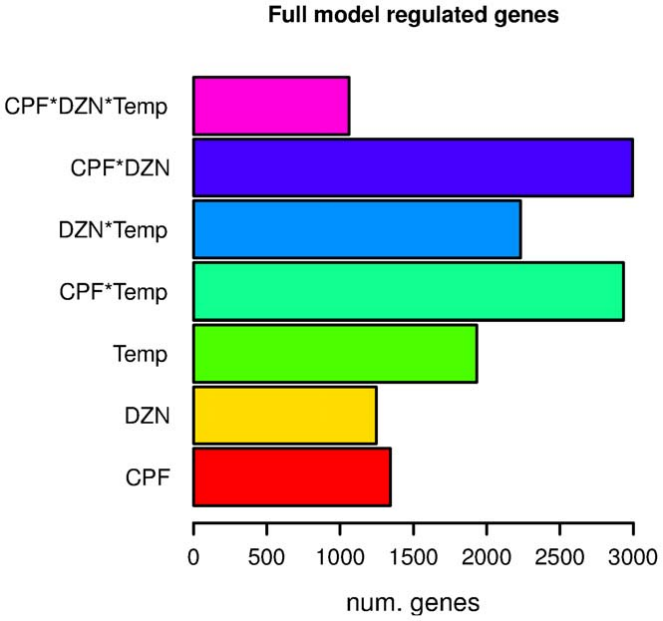
Number of genes significantly regulated by toxicants, temperature and interactions in a full model analysis. We analyzed the transcriptional effect of chlorpyrifos (CPF) and diazinon (DZN), at different temperatures (Temp) using a linear model that considered interactions between environmental factors. In this way, genes significantly affected (−log p-value >2) by CPF, DZN, Temp or any interaction between them (CPF*Temp, DZN*Temp, CPF*DZN*Temp) were identified. Table S8 shows the total numbers and the overlapping genes between variables.

Our analysis of expression profiles at different temperatures revealed little overlap in transcriptional responses to OP. Likewise, the combined treatment (CPF+DZN) results also indicated regulation of different transcriptional responses from what the combinations of single treatment analysis would have predicted. Both results pointed out to gene-by-environment interactions as the underlying cause for expression profiling differences. To study the influence of interactions between toxicants and temperature we re-analyzed all the expression profiles (16°C and 24°C) with a linear model that included the effect of toxicants and temperatures and their interactions. The model included as variables: CPF, DZN, Temperature (Temp), and the interactions: CPF*Temp, DZN*Temp, CPF*DZN and CPF*DZN*Temp. Genes with a −log10 p-value above the threshold were considered significantly influenced by the corresponding variable or interaction (Table S8). Figure 5 shows the significant number of genes per variable. Table S9 also shows the number of overlapping genes between them, since the changes in transcript abundance can be influenced by more than one environmental factor. In summary, 18889 genes were tested of which we found a larger number of genes to be significantly affected by the variables considering the interaction of the toxicants (CFP*DZN, 15.8%) and the interaction of the toxicants with temperature (CPF*Temp, 15.5% and DZN*Temp, 11.8%). The temperature alone and interacting with other factors, affected 87.2% of the differentially expressed genes (6623), while the variables including toxicants affected 72.8% of them. Therefore, the overall influence of temperature on gene expression was larger than of the toxicants as it affected more genes.

We also investigated the biological functions associated to the significant genes per variable. Figures S2, S3 and S4 show the tree distribution of GO terms and the significant terms per treatment (full table and p-values in Table S10). As expected, the analysis showed regulation of monooxygenase activity, lipid transport and metabolic process among others in all categories. Some GO terms were significantly enriched for only one variable. For example, the GO term associated to cellular components of the presynaptic active zone was enriched in the DZN significant genes. More terms were affected only by temperature such as protein folding, dephosphorylation or ATPase activity. Likewise, the domain enrichment analysis indicated overrepresentation of domains related with known responses to OP treatments [4], [28] like detoxification and metabolic transport. Among domains enriched only in one variable we identified ABC transporter-like domains by Temp, CUB-like domains by DZN, or protein of unknown function DUF23 by the interaction CPF*Temp (Table S11).

### Gene-by-environment interactions on detoxification genes

Our first comparison of regulated genes by OP treatments showed a large number of detoxification genes significantly affected by the different treatments and differences in the number of regulated genes (Figure 4 and Figure S1). In detail, we observed a higher percentage of detoxification genes (CYP, SDR, GST, UDP, ABC and NR genes) in the lists of significantly affected genes by DZN (5.2%), Temperature (4.3%), and the interaction CPF*DZN*Temp (4.1%), while lower percentages were observed for the other variables: CPF (3.5%), DZN*Temp (3.4%), CPF*Temp (3.4%) and CPF*DZN (3.6%).

**Figure 6:**
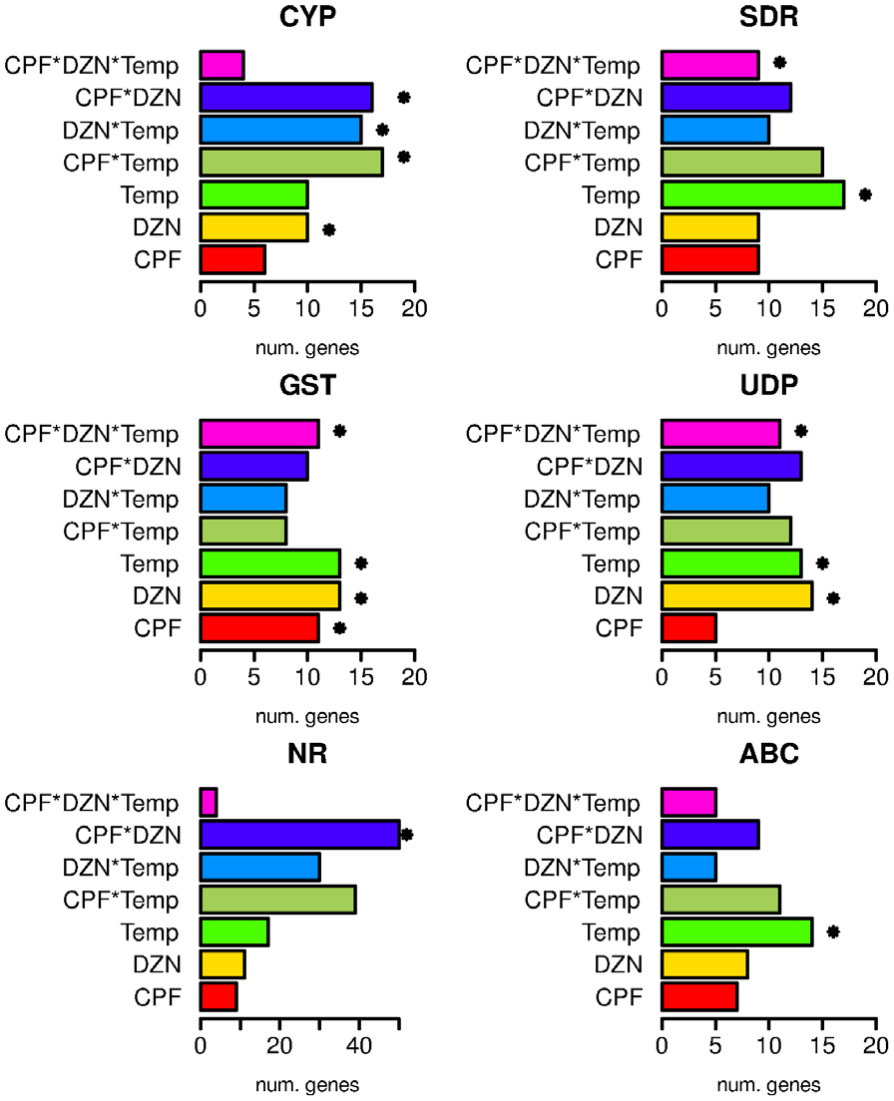
Gene-by-environment interactions on detoxification genes. From the significantly regulated genes in the full model analysis (Figure 5) we selected and plot here the detoxification genes: cytochrome P450 enzymes (CYPs), short chain dehydrogenases (SDR), UDP-glucuronosyl transferases (UGT), glutathione-S-transferases (GST), ABC-transporters (ABC), and nuclear receptor (NR). The full model considered the effect of both toxicants (CPF, DZN) and the temperature (Temp) and any interaction between them (CPF*Temp, DZN*Temp, CPF*DZN*Temp). Asterisks indicate that the domains were significantly enriched in each treatment.

Figure 6 shows the number of detoxification genes regulated by each variable in the linear model. Asterisks in the graph indicated that the number of genes was significantly enriched in a specific group of genes, i.e. considering the number of significant genes by each treatment, the proportion of genes with the domain (e.g. CYP) was statistically relevant. Table S12 shows all the significantly affected detoxification genes, and a summary with CYP genes can be found in Table S7B. In general from all the figures, we would conclude that temperature was a relevant factor in detoxification since it was significantly enriched for ABC, GST, UDP and SDR domains (see also Table 11). Likewise, temperature interaction with CPF and DZN was significantly enriched for CYP and NR, suggesting that a change in temperature may modify the toxicological outcome to OP exposures since their toxicity is determined by oxon production. Also, toxicant interactions (CPF*DZN) revealed a significant enrichment in CYP genes and NR genes, which may be a consequence of binding competition between OP to CYP.

## Discussion

We previously found that CPF and DZN induced dissimilar genes, although they share a similar mode-of-action [28]. Moreover, we showed that the toxicant interactions modified their transcriptional outcome inducing a specific gene transcription response. The effect of temperature treatment was studied by comparing the expression profiles from worms treated with CPF, DZN and a low dose mixture of both at 24°C to the expression profiles from worms treated with identical toxicant concentrations and rearing treatments at 16°C [28]. The gene expression and GO term analysis at 16°C was based on Rank Products method which does not identify the effect of toxicant-temperature interactions. For this purpose the data from the 16°C experiment were re-analyzed together with the data from 24°C. Our results show that there was the lack of commonly regulated genes between the two pesticides at different temperatures. Comparisons across treatments showed that CPF and DZN had also little in common regarding gene expression. Moreover, at 16°C the number of significantly regulated genes was smaller than at 24°C. Yet, the GO terms were able to describe the relative significance of each biological function within the number of significantly regulated genes. The most relevant biological functions affected were similar in all the studies, even when the top significant genes change with a different statistical method [28]. Likewise, the mixture treatments had very different regulated genes to CPF and DZN single treatments. Both results are relevant for toxicological studies, since pesticide classifications are often based on primary enzyme targets, with little reference to non-targets and their effects, such as investigated in this study.

Mixture exposures showed very different transcriptional responses from single toxicants treatments. Our first analysis (Figures 1 to [Fig pone-0024676-g002] [Fig pone-0024676-g003] 4) treated the combined exposure to CPF+DZN as a different independent toxicant treatment. The model attributed the observed gene expression differences to one of the explanatory variables in each model, which were the three toxicant treatments (CPF, DZN and CPF+DZN). It revealed the above mentioned little similarities in transcriptional responses between all the treatments. For example, some biological functions were similarly affected by CPF and the combination, e.g. asymmetric protein localization; while for others the mixture effect was similar to DZN, like ATP binding (Figure 2). Moreover, it showed that the combination may act as a different toxicant affecting different and unique processes, e.g. transcription regulator activity (Figure 2). On the other hand, the full linear model (Figures 5 and 6) allowed us to study gene-by-environment interactions between the different toxicants and temperatures. The full model took into consideration that the combined treatment may have transcriptional responses beyond the single toxicants exposures responses. It attributed the source of gene expression differences to one of the explanatory variables in the model, including interactions. For example, we could determine that in CPF and DZN combined exposures the sequence-specific DNA binding activity (GO:0043565) was affected as a consequence of a toxicant interaction (p-value 1.3303 for CPF*DZN), which was not observed in the single treatments (Table S10). Still, both models complemented each other to reveal the similar and dissimilar biological functions regulated in mixtures treatments compared to single treatments. For instance, the GO term lipid transporter activity (GO:0005319) was significantly enriched in CPF treatment at 16°C and in all treatments at 24°C (Figure 2). In a full model with interactions, the same GO term was enriched in genes affected by temperature, and the interactions between i) CPF*Temp, DZN*Temp and ii) CPF*DZN. The first result suggested that both CPF and DZN had an influence on lipid transport, and that such an influence was temperature dependent. In addition, the second result suggested that the both toxicants affected lipid transport activity in combined treatments as a consequence of toxicants interactions and temperature changes. In summary, both analyses of combined treatments revealed the many differences in gene transcriptional responses to single treatments as a consequence of temperature-toxicants interactions. Moreover, it supported previous results and studies suggesting that transcriptional responses to mixtures may not be defined in terms of antagonistic or synergistic effects, but in terms of interacting or not interacting toxicants.

Temperature affects almost all biological processes, including the transcriptional consequences of toxicants exposure. All the analyses showed an increase in the number of regulated genes at higher temperatures, and a small overlap between toxicants affected genes at different temperatures (Figure 3). As a consequence of the higher number of regulated genes, the single treatments significantly affected more biological processes at higher temperature (Table S3). In addition, the full model indicated that the overall influence of temperature was larger than the toxicants as it affected more genes, by itself or as interacting factor. Therefore, the toxicant modifications on gene expression and on some biological processes were affected by changes in temperature. For example, time of growth and development in *C. elegans* is negatively correlated with temperature. We identified embryonic development (GO:0009790) as a biological function affected by the temperature in the full model analysis. With the same model, affected genes by CPF, DZN and the interaction CPF*DZN*Temp were enriched for the same GO term. However, when the temperature effect was not considered this GO term was significantly enriched at 24°C for all treatments, but not at lower temperature. Because *C. elegans* develops faster at higher temperature, the toxicant exposure had a stronger influence on developmental genes at high temperature than it had a 16°C. Likewise, we observed that temperature modified the expression of many other genes affected by the toxicants (CPF*Temp and DZN*Temp interactions). This was in agreement with toxicological studies in other species showing increased effect of both toxicants with an increased temperature [11], [12], [30]. In this regard, increased metabolic uptake and increased toxicant accumulation has been considered a main cause for increased toxicant effect with temperature. At the transcriptional level, we observed a similar response with an increase in the number of significantly affected genes and biological processes at higher temperatures as a result of toxicant-temperatures interactions.

Temperature affected detoxification genes in single and combined treatments independently from the OP effects. One of the few common regulated processes at all temperatures was associated to detoxification genes; however, not all detoxification genes had the same response to temperature and toxicants interactions. The analysis of commonly regulated genes at different temperatures (Figure 3 and Tables S5 and S6) indicated that CYP genes were regulated by all the toxicants independently from temperature. In that sense, the full model indicated significant effect on CYP genes expression by DZN, and the interactions between CPF*Temp, DZN*Temp and CPF*DZN. In other words, toxicants exposures were necessary to trigger CYP genes expression differences and the temperature changes were not enough to explain and possibly induce those differences. Indeed, a group of genes seemed to have specificity in their responses to either CPF or DZN, with weakly or no influence by temperature (Table S5).

From gene expression results it is difficult to evaluate the antagonistic or synergistic effects between toxicants because the regulation of different genes buffers the effects of increased toxicant concentrations and interacting effects. Therefore, antagonistic or synergistic concepts have a lack of meaning to describe toxicants interactions at gene expression level. Yet, they allow for analyzing toxicant effects on specific biological functions. An example was shown for detoxification genes (Figures 4 and 6). In general, more detoxification genes were regulated in the mixture treatments suggesting a synergistic effect. Logic dictates that because the worms were exposed to higher concentrations of both toxicants, more enzymatic activity was required to detoxify the chemicals. However, a similar principle should apply in DZN treatments vs. CPF treatment, and this was not the case. At lower temperature, all the treatments regulated similar numbers of CYP, SDR and UDP, while at higher temperatures CPF and DZN had similar numbers of SDR and UDP, but very different numbers of CYP, NR or GST genes. These results suggest that at a lower temperature, the toxicant concentration was less relevant to explain the number of regulated detoxification genes; but that at higher temperature, the higher chemical concentration induced a higher interacting effect of the chemicals on some genes.

In conclusion, we analyzed the transcriptional responses to interacting environmental stressors. Three factors were considered: the toxicants CPF and DZN, and temperature. We focused our analysis on detoxification genes because toxicity on OP can be partially explained by bioactivation of OP to highly toxic oxon forms mediated by detoxification enzymes. Our results indicated that the expression of detoxification genes is modulated by the interaction of toxicants. On the other hand, we showed that the interaction between temperature and toxicants has a major effect on the expression of detoxification and other genes. Next to temperature, the interaction with the genetic background is another mode by which the effects of toxicants on gene expression can be modified. We showed that gene expression is variable during aging and is affected by genotype by age interactions [31]. It is likely that the toxicant effects on gene expression are not only determined by dosage/environment, but also by genotype and age and the interaction between those factors. Temperature had a strong effect on transcript abundance as it affected the expression of many genes. Moreover, a larger number of detoxification genes were significantly regulated by the interactions between temperature and the toxicants than by the OP alone. Indeed, it has been shown that toxicity of OP increased with temperatures [11], [12]; accordingly, our results suggest that higher toxicity of OP with temperature is a consequence of gene-environment interactions on detoxification genes. This is especially important when evaluating and translating the effects of altered gene expression profiles to higher organization levels. For instance by relating the genetic control of body size or other complex traits to the population level in disturbed environments [32], [33]. Finally, it shows that many effects on gene expression of combination of treatments cannot be deduced by combining the results from the single treatments.

## Methods

### *C. elegans* culturing

The Bristol N2 strain was cultured on standard nematode growth medium (NGM) with *E. coli* OP50 as food source. Nematodes were bleached (0.5 M NaOH, 1% hypochlorite) and eggs were collected and inoculated in (9 cm diameter) dishes with the toxicants. After 40 hours at 24°C, late L3 stage nematodes were collected, frozen in liquid nitrogen and kept at −80°C until the RNA extraction procedure [28]. This developmental stage was the same for worms reared at 16°C and harvested for RNA after 72 hours [28], [31].

### Toxicant treatments

Because we aimed to study the gene transcriptional effects of exposure to the pesticides at levels that did not affect development or reproduction of the worms, we focused on pesticide concentrations below the levels that elicit a clear toxic effect. The selection of the test concentrations was based on the EC50 levels of CPF and DZN for reproduction. In previous studies using *C. elegans*, EC50 values for CPF differed among different experiments (same culture media) (EC50 = 3.5 mg/L [34] and 0.9–1.3 mg/L [35]). Based on these data we selected 2 mg/L as a reasonable value for the EC50 of CPF. For DZN, a much larger variation of EC50 values was found [34], ranging from (2.8 mg/L to 203 mg/L). In order to prevent having DZN levels affecting the worms, we chose 4 mg/L as a reasonable value for the EC50 of DZN. We then decided to analyze gene expression in response to the toxicants concentrations a factor 4 below the EC50 values for CPF and DZN. We expected that no developmental effects would occur at these levels, whereas the exposure levels were thought to be high enough to affect gene expression. In line with our expectations, we did not observe any developmental effects which is in agreement with previous studies showing that, at a factor 4 below the EC50, no observable sublethal effects were recorded [36], [37].

The concentrations were 0.5 mg/l of CPF (Cyren®/Nufos®, Cheminova A/S [Lemvig, Denmark]) and 1.0 mg/l of DZN (Supelco [Bellefonte, Pennsylvania 16823, USA]).

The combination of the two OP contained the exact sum of both single concentrations (DZN [1 mg/L] and CPF [0.5 mg/L]). Both the compounds CPF and DZN were chemically stable throughout our experimental periods. CPF has been shown to be highly stable for weeks in many different environments [38]. That is also one of the reasons why Svendsen *et al*. [35] used the same compound in chronic *C. elegans* studies. Also DZN is a very stable compound [39]. No degradation occurs within the exposure periods of a few days we used. Based on the EC50 values, the combination was equitoxic for both compounds. Here we defined equitoxic as the situation where the Toxic Units (TU) of the two compounds are equal. A TU  =  (concentration of compound)/EC50_compound_ [9], [10]. We observed that CPF treated worms were slightly less mobile compared to the control, but this effect was very mild and hardly noticeable. The experiment started with eggs placed on NGM dishes with the toxicants and *E. coli* OP50 as food source. After 40 hours at 24 °C, worms from 4 petri-dishes were collected as one sample. A total of 6 replicates per treatment were collected (24 petri dishes), and immediately frozen in liquid nitrogen until RNA extraction. All OP were dissolved in acetone and added to 10 ml of NGM poured in each 9 cm petri dish used for the culture. Nematodes without treatments were grown simultaneously with similar concentrations of acetone in a control culture.

### Microarrays

RNA from nematodes was extracted following the Trizol method, and the RNeasy Micro kit (Quiagen, Valencia, CA, USA) was used to clean up the samples. Labeled cDNA was produced with the kit Array 900 HS from Genisphere and Superscript II from Invitrogen. The 60-mers arrays were purchased by Washington University and they were hybridized following the Genisphere Array 900 HS protocol with modifications. Extracts from CPF, DZN and the CPF/DZN combination exposures were hybridized with the control samples in each array. Six independent biological replicates were used per treatment to produce six replicated microarrays per experiment in a dye-swap design.

All microarray raw data and normalized data have been deposited in Gene Expression Omnibus (www.ncbi.nlm.nih.gov/geo/), a MIAME compliant database, with the accession number GSE24257. Microarray platform information number in GEO: GPL4038. Expression profiles of *C. elegans* exposure at 16°C were downloaded from GEO (GSE16719).

### Microarray Analysis

A Perking & Elmer scanner was used to extract the raw intensities from the microarrays. Preprocessing and normalization of all microarrays (16°C and 24°C) were done in the R software [40] using Limma package [41]. The Loess method [42] was used for normalization within arrays and normalization between arrays was done using aquantile method [43], both of them are included in the Limma package for R. Outliers from all experiments were identified using a linear model per toxicant. The models fit log2 expression values according to the toxicant treatment (CPF, DZN or CPF+DZN) at two temperatures (16°C and 24°C) and remove values outside the 0.995 confidence interval, one spot at the time, recursively. No more than 6 values were allowed to be removed.

To identify the differentially expressed genes in each treatment we used linear models per toxicant and temperature (gene expression  =  Toxicant (effect) + error). The lm function in R stats package was used to implement the linear models analysis with recommended default options [40]. For threshold determination we used a permutation approach. For each of the 23,232 permutations used we randomly picked a transcript (array spot), which could only be picked once. We combined all the expression values of this transcript and randomly distributed them over the replicates and used them in the linear model. In this way we obtained a threshold for each of the toxicants. We used a −log10 p-value 2 as common threshold for the analysis, which resembles to the following FDR per toxicant: 0.0155 for CPF at 24°C, 0.0148 for DZN at 24°C, 0.0168 for CPF+DZN at 24°C, 0.0142 for CPF at 16°C, 0.0151 for DZN at 16°C, and 0.0148 for CPF+DZN, at 16°C.

To estimate the influence of interacting environmental factors (toxicants and temperature) we used a full linear model (lm function in R) with all the toxicants and both temperatures as influential variables for gene expression (gene expression  =  CPF (effect) * DZN (effect) * Temperature (effect) + error). Therefore, we were able to estimate the effect of each factor on the measured variation in gene expression as well as the effect of any possible interaction between them. For threshold determination we used similar permutation approach as above. We also used a −log10 p-value 2 as common threshold, which resembles to the following FDR per variable: 0.0096 for CPF, 0.0098 for DZN, 0.0098 for Temp, 0.0098 for CPF*Temp, 0.0098 for DZN*Temp, 0.0102 for CPF*DZN, 0.0099 for CPF*DZN*Temp.

### Detoxification genes

We identified genes involved in detoxification based on protein domains. For each category of enzymes or proteins referred in the text (CYP, SDR, UDP, GST, ABC) we selected functional domains related to their function in INTERPRO (www.ebi.ac.uk/interpro/). Information of functional domains in *C. elegans* genes was downloaded from Wormbase [44] using the last stable release WB195. In this way, we assumed a gene with a domain, predicted or confirmed, related to detoxification is a candidate gene to be involved in OP metabolism.

Domain IDs used were: CYP (PF00067, IPR001128, IPR002397, IPR002401, IPR002402, IPR002403); SDR (PF00106, IPR002198); UDP (PF00201, IPR002213); GST (IPR010987, IPR004045, IPR004046, PF02798, IPR005442, IPR003082, PF00043); ABC (IPR003439, IPR011527, PF00005, IPR010509, PF06472).

Nuclear Receptors were selected from literature [45].

### Enrichment analysis

Gene Ontology (GO) data and functional domain data were extracted also from Wormbase release WB195 [44]. GO terms and domains with less than 4 genes were discarded. Over-represented groups of GO terms and domains were identified using a hypergeometric test (P-value <0.05) with the R function phyper form the R basic package stats [40]. In this way we analyzed 396 unique GO terms and 1003 unique INTERPRO id numbers, from 16,947 and 8682 annotated genes, respectively. R packages topGO [46], GO.db [47], annotate [48] and Rgraviphz [49] were used to produce Figures S2, S3 and S4.

## Supporting Information

Figure S1 **Percentage of the total number of detoxification genes regulated by treatment and temperature.**
(PDF)

Figure S2 **Molecular Function GO tree representation for significantly enriched terms and their parents.** Color indicated enrichment in each treatment. The figure is followed by a table with GO terms ID and description for the enriched terms and a table with all the GO terms ID and description in the figure.
(PDF)

Figure S3 **Biological Process GO tree representation for significantly enriched terms and their parents.** Color indicated enrichment in each treatment. The figure is followed by a table with GO terms ID and description for the enriched terms and a table with all the GO terms ID and description in the figure.
(PDF)

Figure S4 **Cellular Component GO tree representation for significantly enriched terms and their parents.** Color indicated enrichment in each treatment. The figure is followed by a table with GO terms ID and description for the enriched terms and a table with all the GO terms ID and description in the figure.
(PDF)

Table S1 **Significantly affected genes by each of the analyzed toxicants and two temperatures.** Each worksheet contains the list of affected genes by each treatment.
(XLS)

Table S2 **List of commonly regulated genes by all toxicants treatments at each temperature: 10 genes at 16°C and 53 at 24°C (** **Figure 1** **).**
(DOC)

Table S3 **List of enriched Gene Ontology (GO) terms in regulated genes by toxicants at different temperatures (from** **Figure 1** **).** GO data were extracted from Wormbase release WB195. First column shows the GO ID number. Next six columns show the −log10 p-value for each treatment. The description of the GO term is also shown. Cells in yellow indicate significant GO terms (p-value <0.05 or −log10 p-value >1.3, hypergeometric test)
(XLS)

Table S4 **List of enriched functional domains in regulated genes by toxicants at different temperatures (from** **Figure 1** **).** Domains associations were extracted from Wormbase release WB195. First column shows the INTERPRO ID number. Next six columns show the −log10 p-value for each treatment. The description of the domain term is also shown. Cells in yellow indicate significant domains (p-value <0.05 or −log10 p-value >1.3, hypergeometric test)
(XLS)

Table S5 **List of commonly regulated genes per toxicant at both temperatures: 75 genes for CPF, 72 genes for DZN, and 112 genes for CPF+DZN (** **Figure 3** **).**
(DOC)

Table S6 **List of enriched functional domains in regulated genes by toxicants at different temperatures (from** **Figure 3** **and Table S5).**
(DOC)

Table S7 **List of significantly regulated cytochrome P450 genes by CPF, DZN and temperature.**
(DOC)

Table S8 **Significantly affected genes in the full model analysis (** **Figure 5** **).** Each worksheet contains the list of affected genes by each factor: chlorpyrifos (CPF), diazinon (DZN), temperature (Temp); and the interactions of them: CPF*Temp, DZN*Temp, CPF*DZN, CPF*DZN*Temp.
(XLS)

Table S9 **Number of significantly expressed genes per variable in the full model (** **Figure 5** **) and the overlap between variables.** Numbers of gene per variable are indicated in bold (diagonal). Since a gene may be affected by more than one variable, the overlap between variables is also indicated. The last row shows the number of genes significantly affected by only one variable.
(DOC)

Table S10 **List of enriched Gene Ontology (GO) terms in regulated genes by toxicants at different temperatures in the full model analysis (from** **Figure 5** **).** GO data were extracted from Wormbase release WB195. First column shows the GO ID number. Next six columns show the −log10 p-value for each treatment. The description of the GO term is also shown. Cells in yellow indicate significant GO terms (p-value <0.05 or −log10 p-value >1.3, hypergeometric test)
(XLS)

Table S11 **List of enriched functional domains in regulated genes by toxicants at different temperatures in the full model analysis (from** **Figure 5** **).** Domains associations were extracted from Wormbase release WB195. First column shows the INTERPRO ID number. Next six columns show the −log10 p-value for each treatment. The description of the domain term is also shown. Cells in yellow indicate significant domains (p-value <0.05 or −log10 p-value >1.3, hypergeometric test)
(XLS)

Table S12 **List of significantly regulated detoxification genes by CPF, DZN and temperature.** Worksheet *Single models* include all the genes predicted to code for CYP, SDR, GST or UDP domains and significantly affected by at least one of the treatments using a model that only considered independent effects. Worksheet *Full model* include all the genes predicted to code for CYP, SDR, GST or UDP domains and significantly affected by at least one of the treatments using a model that considered interacting effects.
(XLS)

## References

[1] Pope CN (1999). Organophosphorus Pesticides: They all have the same mechanism of toxicity?. Journal of Toxicology and Environmental Health Part B: Critical Reviews.

[2] Slotkin TA, MacKillop EA, Ryde IT, Tate CA, Seidler FJ (2007). Screening for developmental neurotoxicity using PC12 cells: Comparisons of organophosphates with a carbamate, an organochlorine, and divalent nickel.. Environmental Health Perspectives.

[3] Slotkin TA, Seidler FJ (2007). Comparative developmental neurotoxicity of organophosphates in vivo: Transcriptional responses of pathways for brain cell development, cell signaling, cytotoxicity and neurotransmitter systems.. Brain Research Bulletin.

[4] Lewis J, Szilagyi M, Gehman E, Dennis W, Jackson D (2009). Distinct patterns of gene and protein expression elicited by organophosphorus pesticides in *Caenorhabditis elegans*.. BMC Genomics.

[5] Hook SE, Skillman AD, Gopalan B, Small JA, Schultz IR (2008). Gene expression profiles in rainbow trout, *Onchorynchus mykiss,* exposed to a simple chemical mixture.. Toxicol Sci.

[6] Mumtaz MM, Tully DB, El-Masri HA, De Rosa CT (2002). Gene induction studies and toxicity of chemical mixtures.. Environmental Health Perspectives.

[7] Jonker MJ, Svendsen C, Bedaux JJM, Bongers M, Kammenga JE (2005). Significance testing of synergistic/antagonistic, dose level-dependent, or dose ratio-dependent effects in mixture dose-response analysis.. Environmental Toxicology and Chemistry.

[8] Spurgeon DJ, Jones OAH, Dorne J-LCM, Svendsen C, Swain S (2010). Systems toxicology approaches for understanding the joint effects of environmental chemical mixtures.. Science of The Total Environment.

[9] Jonker MJ, Piskiewicz AM, Castellà NII, Kammenga JE (2004). Toxicity of binary mixtures of cadmium-copper and carbendazim-copper to the nematode *Caenorhabditis elegans.*. Environmental Toxicology and Chemistry.

[10] Jonker MJ, Sweijen RAJC, Kammenga JE (2004). Toxicity of simple mixtures to 14. the nematode *Caenorhabditis elegans* in relation to soil sorption.. Environmental Toxicology and Chemistry.

[11] Osterauer R, Köhler H-R (2008). Temperature-dependent effects of the pesticides thiacloprid and diazinon on the embryonic development of zebrafish (*Danio rerio*).. Aquatic Toxicology.

[12] De Silva PMCS, Pathiratne A, van Gestel CAM (2009). Influence of temperature and soil type on the toxicity of three pesticides to *Eisenia andrei.*. Chemosphere.

[13] Bindesbøl AM, Bayley M, Damgaard C, Holmstrup M (2009). Impacts of heavy metals, polyaromatic hydrocarbons, and pesticides on freeze tolerance of the earthworm *Dendrobaena octaedra*.. Environmental Toxicology and Chemistry.

[14] Poet TS, Wu H, Kousba AA, Timchalk C (2003). In vitro rat hepatic and intestinal metabolism of the organophosphate pesticides chlorpyrifos and diazinon.. Toxicol Sciences.

[15] Eaton DL, Daroff RB, Autrup H, Bridges J, Buffler P (2008). Review of the toxicology of chlorpyrifos with an emphasis on human exposure and neurodevelopment.. Critical Reviews in Toxicology 38:.

[16] Reichert K, Menzel R (2005). Expression profiling of five different xenobiotics using a *Caenorhabditis elegans* whole genome microarray.. Chemosphere.

[17] Menzel R, Rödel M, Kulas J, Steinberg CEW (2005). CYP35: Xenobiotically induced gene expression in the nematode *Caenorhabditis elegans*.. Archives of Biochemistry and Biophysics.

[18] Roh J-Y, Jung I-H, Lee J-Y, Choi J (2007). Toxic effects of di(2-ethylhexyl)phthalate on mortality, growth, reproduction and stress-related gene expression in the soil nematode *Caenorhabditis elegans*.. Toxicology.

[19] Cui Y, McBride S, Boyd W, Alper S, Freedman J (2007). Toxicogenomic analysis of *Caenorhabditis elegans* reveals novel genes and pathways involved in the resistance to cadmium toxicity.. Genome Biology.

[20] Kulas J, Schmidt C, Rothe M, Schunck W-H, Menzel R (2008). Cytochrome P450-dependent metabolism of eicosapentaenoic acid in the nematode *Caenorhabditis elegans*.. Archives of Biochemistry and Biophysics.

[21] Holmstrup M, Bindesbøl A-M, Oostingh GJ, Duschl A, Scheil V (2010). Interactions between effects of environmental chemicals and natural stressors: A review.. Science of The Total Environment.

[22] Scheil V, Köhler H-R (2009). Influence of nickel chloride, chlorpyrifos, and imidacloprid in combination with different temperatures on the embryogenesis of the zebrafish *Danio rerio*.. Archives of Environmental Contamination and Toxicology.

[23] Gillooly JF, Brown JH, West GB, Savage VM, Charnov EL (2001). Effects of size and temperature on metabolic rate.. Science.

[24] Sørensen JG, Nielsen MM, Kruhøffer M, Justesen J, Loeschcke V (2005). Full genome gene expression analysis of the heat stress response in *Drosophila melanogaster*.. Cell Stress and Chaperones.

[25] Lee S-J, Kenyon C (2009). Regulation of the longevity response to temperature by thermosensory neurons in *Caenorhabditis elegans*.. Current Biology.

[26] Nota B, Van Straalen NM, Ylstra B, Roelofs D (2010). Gene expression microarray analysis of heat stress in the soil invertebrate *Folsomia candida*.. Insect Molecular Biology.

[27] Li Y, Alvarez OAA, Gutteling EW, Tijsterman M, Fu JJ (2006). Mapping determinants of gene expression plasticity by genetical genomics in *C. elegans*.. Plos Genetics.

[28] Viñuela A, Snoek LB, Riksen JAG, Kammenga JE (2010). Genome-wide gene expression analysis in response to organophosphorus pesticide chlorpyrifos and diazinon in *C. elegans*.. PLoS ONE.

[29] Lindblom TH, Dodd AK (2006). Xenobiotic detoxification in the nematode *Caenorhabditis elegans*.. Journal of Experimental Zoology Part A: Comparative Experimental Biology.

[30] Lydy MJ, Belden JB, Ternes MA (1999). Effects of temperature on the toxicity of M-parathion, chlorpyrifos, and pentachlorobenzene to *Chironomus tentans*.. Archives of Environmental Contamination and Toxicology.

[31] Viñuela A, Snoek LB, Riksen JAG, Kammenga JE (2010). Genome-wide gene expression regulation as a function of genotype and age in *C. elegans*.. Genome Research.

[32] Kammenga JE, Doroszuk A, Riksen JAG, Hazendonk E, Spiridon L (2007). A *Caenorhabditis elegans* wild type defies the temperature-size rule owing to a single nucleotide polymorphism in *tra-3*.. PLoS Genetics.

[33] Spurgeon DJ, Svendsen C, Weeks JM, Hankard PK, Stubberud HE (2003). Quantifying copper and cadmium impacts on intrinsic rate of population increase in the terrestrial oligochaete *Lumbricus rubellus*.. Environmental Toxicology and Chemistry.

[34] Martin HL, Svendsen C, Lister LJ, Gomez-Eyles JL, Spurgeon DJ (2009). Measurement and modelling of the toxicity of binary mixtures in the nematode *Caenorhabditis elegans* - A test of independent action.. Environmental Toxicology and Chemistry.

[35] Svendsen C, Siang P, Lister LJ, Rice A, Spurgeon DJ (2010). Similarity, independence, or interaction for binary mixture effects of nerve toxicants for the nematode *Caenorhabditis elegans*.. Environmental Toxicology and Chemistry.

[36] Rajini PS, Melstrom P, Williams PL (2008). A comparative study on the relationship between various toxicological endpoints in *Caenorhabditis elegans* exposed to organophosphorus insecticides.. Journal of Toxicology and Environmental Health, Part A,.

[37] Boyd WA, Smith MV, Kissling GE, Freedman JH (2010). Medium- and high-throughput screening of neurotoxicants using *C. elegans*.. Neurotoxicology and Teratology.

[38] Fang H, Yu YL, Wang XG, Chu XQ, Pan XD (2008). Effects of repeated applications of chlorpyrifos on its persistence and soil microbial functional diversity and development of its degradation capability.. Bulletin of Environmental Contamination and Toxicology.

[39] Drezina B, Stegu M (2007). Degradation study of selected organophosphorus insecticides in natural waters.. International Journal of Environmental Analytical Chemistry 87:.

[40] Computing RFfS, Team RDC (2011). R: A language and environment for statistical computing..

[41] Smyth GK (2005). Limma: Linear models for microarray data. Bioinformatics and Computational Biology Solution Using R and Bioconductor..

[42] Smyth GK, Speed T (2003). Normalization of cDNA microarray data.. Methods.

[43] Yang YH, Thorne NP, Goldstein DR (2003). Normalization for two-color cDNA microarray data.. Science and Statistics: A Festschrift for Terry Speed.

[44] Stein L, Sternberg P, Durbin R, Thierry-Mieg J, Spieth J (2001). WormBase: network access to the genome and biology of Caenorhabditis elegans.. Nucleic Acids Research.

[45] Antebi A, WormBook (2006). Nuclear hormone receptors in *C. elegans.*. http://www.wormbook.org.

[46] Alexa A, Rahnenführer J, Lengauer T (2006). Improved scoring of functional groups from gene expression data by decorrelating GO graph structure Bioinformatics..

[47] Carlson M, Falcon S, Pages H, Li N (2007). GO.db: A set of annotation maps describing the entire Gene Ontology.. R package version.

[48] Gentleman R (2003). Annotate: Annotation for microarrays.. R package version.

[49] Gentry J, Long L, Gentleman R, Falcon S, Hahne F (2010). Rgraphviz: Provides plotting capabilities for R graph objects.. R package version.

