# Supplementary figures and images for: Gene Expression Modifications by Temperature-Toxicants Interactions in Caenorhabditis elegans
Source: PLoS One. 2011 Sep 9;6(9):e24676. doi: 10.1371/journal.pone.0024676 (PMC3170376; doi:10.1371/journal.pone.0024676)

# % detoxification genes

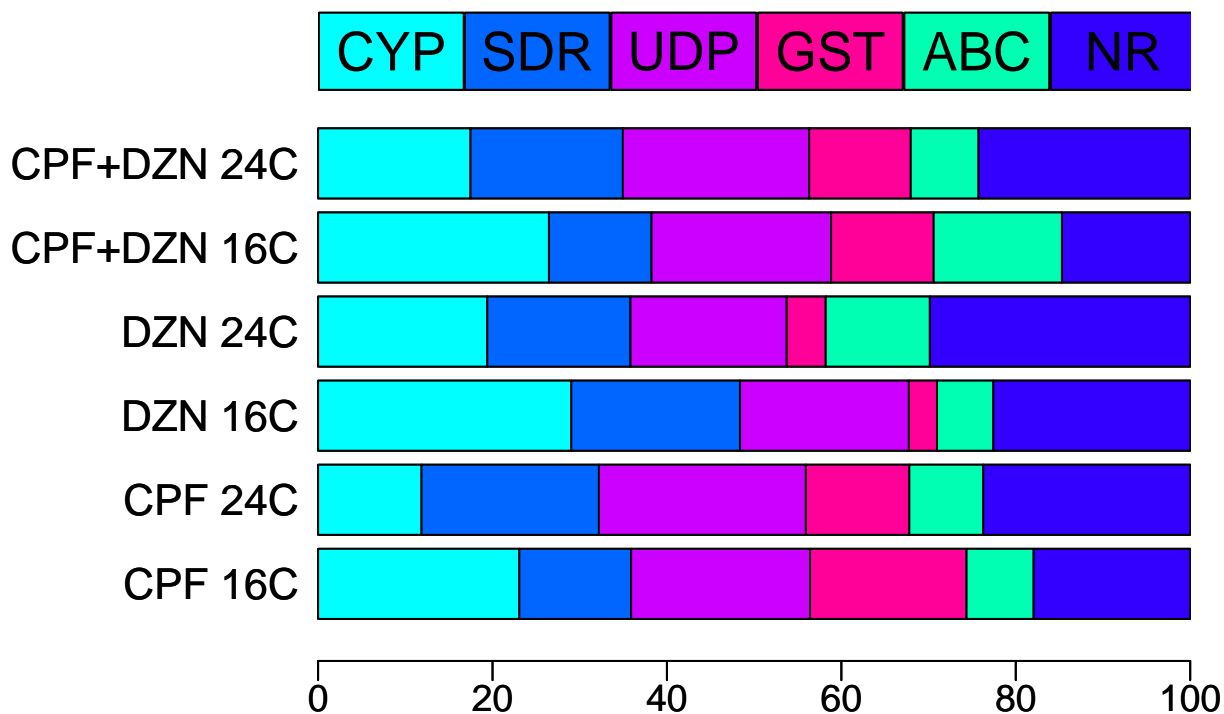

Supplement: Figure S1 — Percentage of the total number of detoxification genes regulated by treatment and temperature. (PDF) [file pone.0024676.s001.pdf]
