# Supplementary material for: Gene Expression Modifications by Temperature-Toxicants Interactions in Caenorhabditis elegans
Source: PLoS One. 2011 Sep 9;6(9):e24676. doi: 10.1371/journal.pone.0024676 (PMC3170376; doi:10.1371/journal.pone.0024676)
Supplement: Figure S2 — Molecular Function GO tree representation for significantly enriched terms and their parents. Color indicated enrichment in each treatment. The figure is followed by a table with GO terms ID and description for the enriched terms and a table with all the GO terms ID and description in the figure. (PDF) [file pone.0024676.s002.pdf]

Molecular Function (GO:0003674)

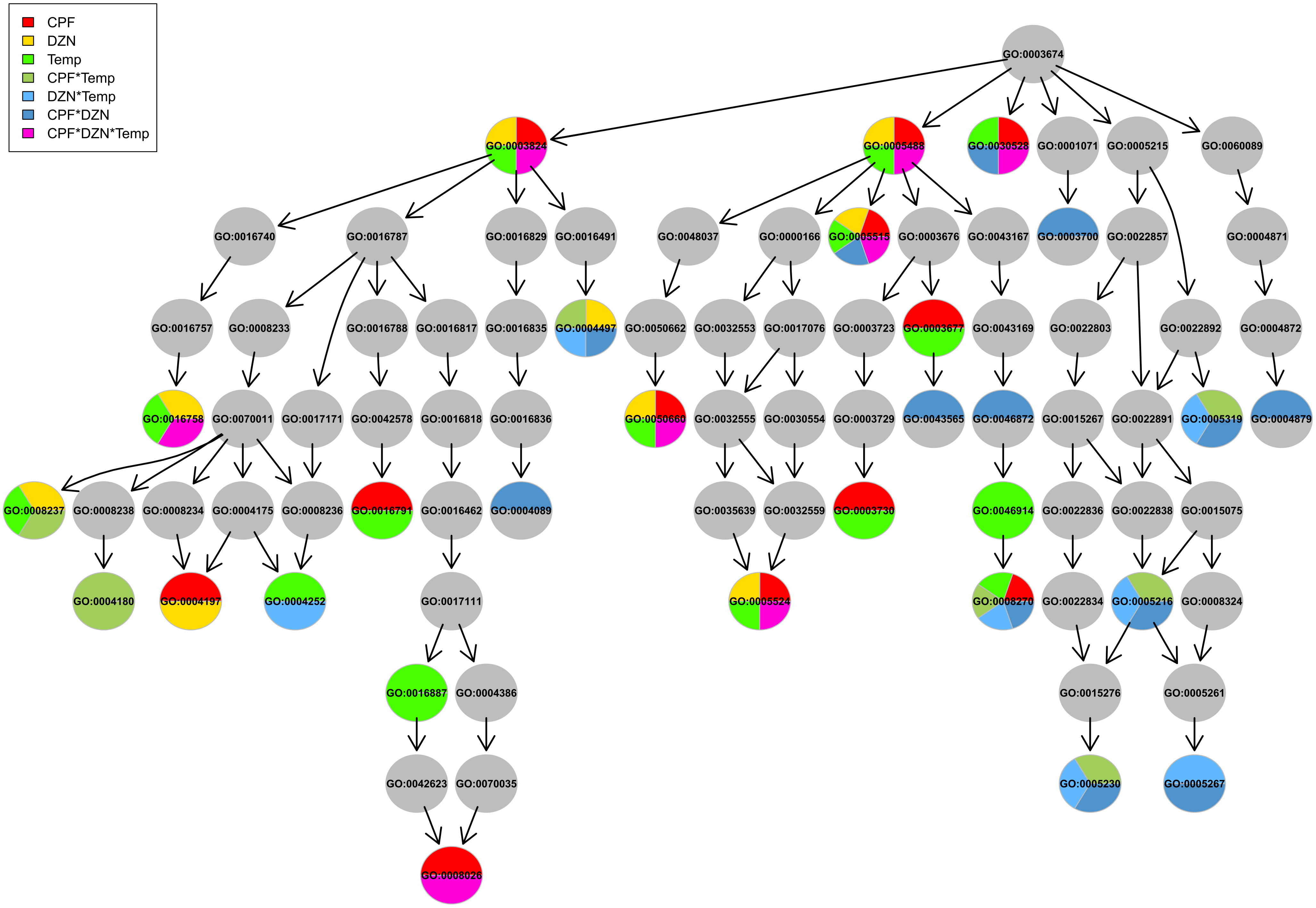

Significant GO terms in at least one treatment	
**Go Term ID**	**Description**
GO:0003677	DNA binding
GO:0003730	mRNA 3'-UTR binding
GO:0003824	catalytic activity
GO:0004197	cysteine-type endopeptidase activity
GO:0005488	binding
GO:0005515	protein binding
GO:0005524	ATP binding
GO:0008026	ATP-dependent helicase activity
GO:0008270	zinc ion binding
GO:0016791	phosphatase activity
GO:0030528	transcription regulator activity
GO:0050660	flavin adenine dinucleotide binding
GO:0004497	monooxygenase activity
GO:0008237	metallopeptidase activity
GO:0016758	transferase activity, transferring hexosyl groups
GO:0004252	serine-type endopeptidase activity
GO:0016887	ATPase activity
GO:0046914	transition metal ion binding
GO:0004180	carboxypeptidase activity
GO:0005216	ion channel activity
GO:0005230	extracellular ligand-gated ion channel activity
GO:0005319	lipid transporter activity
GO:0005267	potassium channel activity
GO:0003700	sequence-specific DNA binding transcription factor activity
GO:0004089	carbonate dehydratase activity
GO:0004879	ligand-dependent nuclear receptor activity
GO:0043565	sequence-specific DNA binding
GO:0046872	metal ion binding

Go Term ID	Description		Go Term ID	Description
GO:0003677	DNA binding		GO:0016836	hydro-lyase activity
GO:0003730	mRNA 3'-UTR binding		GO:0017111	nucleoside-triphosphatase activity
GO:0003824	catalytic activity		GO:0022838	substrate-specific channel activity
GO:0004197	cysteine-type endopeptidase activity		GO:0022892	substrate-specific transporter activity
GO:0005488	binding		GO:0032559	adenyl ribonucleotide binding
GO:0005515	protein binding		GO:0035639	purine ribonucleoside triphosphate binding
GO:0005524	ATP binding		GO:0042578	phosphoric ester hydrolase activity
GO:0008026	ATP-dependent helicase activity		GO:0042623	ATPase activity, coupled
GO:0008270	zinc ion binding		GO:0043169	cation binding
GO:0016791	phosphatase activity		GO:0050662	coenzyme binding
GO:0030528	transcription regulator activity		GO:0070011	peptidase activity, acting on L-amino acid peptides
GO:0050660	flavin adenine dinucleotide binding		GO:0070035	purine NTP-dependent helicase activity
GO:0004497	monooxygenase activity		GO:0003723	RNA binding
GO:0008237	metallopeptidase activity		GO:0004386	helicase activity
GO:0016758	transferase activity, transferring hexosyl groups		GO:0004871	signal transducer activity
GO:0004252	serine-type endopeptidase activity		GO:0005215	transporter activity
GO:0016887	ATPase activity		GO:0008233	peptidase activity
GO:0046914	transition metal ion binding		GO:0008324	cation transmembrane transporter activity
GO:0004180	carboxypeptidase activity		GO:0015267	channel activity
GO:0005216	ion channel activity		GO:0016462	pyrophosphatase activity
GO:0005230	extracellular ligand-gated ion channel activity		GO:0016740	transferase activity
GO:0005319	lipid transporter activity		GO:0016788	hydrolase activity, acting on ester bonds
GO:0005267	potassium channel activity		GO:0016835	carbon-oxygen lyase activity
GO:0003700	sequence-specific DNA binding transcription factor activity		GO:0017171	serine hydrolase activity
GO:0004089	carbonate dehydratase activity		GO:0022834	ligand-gated channel activity
GO:0004879	ligand-dependent nuclear receptor activity		GO:0022891	substrate-specific transmembrane transporter activity
GO:0043565	sequence-specific DNA binding		GO:0030554	adenyl nucleotide binding
GO:0046872	metal ion binding		GO:0032555	purine ribonucleotide binding
GO:0001071	nucleic acid binding transcription factor activity		GO:0043167	ion binding
GO:0003674	molecular_function		GO:0048037	cofactor binding
GO:0003676	nucleic acid binding		GO:0016787	hydrolase activity
GO:0003729	mRNA binding		GO:0016757	transferase activity, transferring glycosyl groups
GO:0004175	endopeptidase activity		GO:0016829	lyase activity
GO:0004872	receptor activity		GO:0017076	purine nucleotide binding
GO:0005261	cation channel activity		GO:0022803	passive transmembrane transporter activity

GO:0008234	cysteine-type peptidase activity		GO:0022836	gated channel activity
GO:0008236	serine-type peptidase activity		GO:0022857	transmembrane transporter activity
GO:0008238	exopeptidase activity		GO:0032553	ribonucleotide binding
GO:0015075	ion transmembrane transporter activity		GO:0060089	molecular transducer activity
GO:0015276	ligand-gated ion channel activity		GO:0000166	nucleotide binding
GO:0016491	oxidoreductase activity		GO:0016817	hydrolase activity, acting on acid anhydrides
GO:0016818	hydrolase activity, acting on acid anhydrides, in phosphorus-containing anhydrides			
