# Supplementary material for: Gene Expression Modifications by Temperature-Toxicants Interactions in Caenorhabditis elegans
Source: PLoS One. 2011 Sep 9;6(9):e24676. doi: 10.1371/journal.pone.0024676 (PMC3170376; doi:10.1371/journal.pone.0024676)
Supplement: Figure S3 — Biological Process GO tree representation for significantly enriched terms and their parents. Color indicated enrichment in each treatment. The figure is followed by a table with GO terms ID and description for the enriched terms and a table with all the GO terms ID and description in the figure. (PDF) [file pone.0024676.s003.pdf]

Biological Process (GO:0008150)

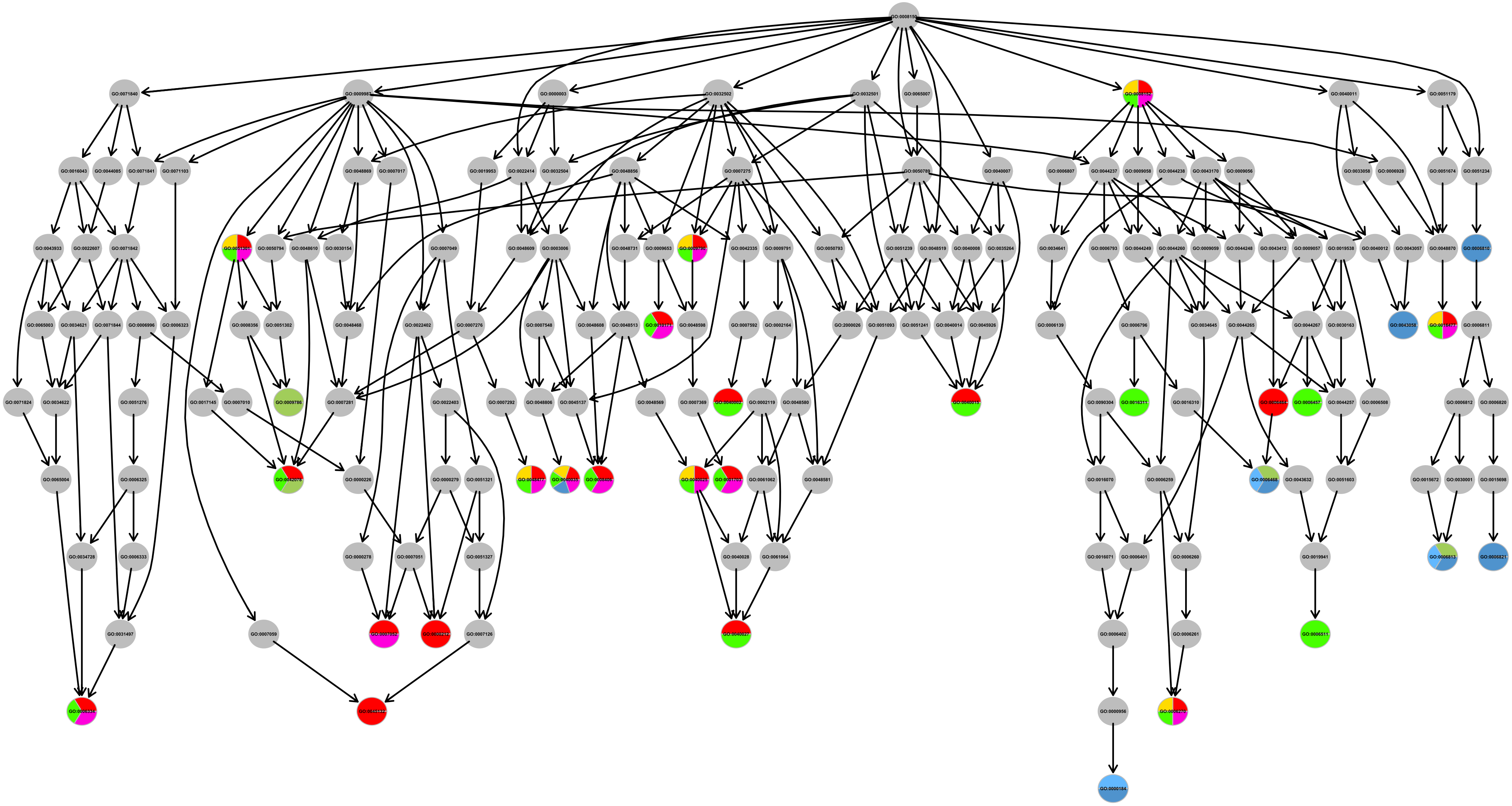

Significant GO terms in at least one treatment	
**Go Term ID**	**Description**
GO:0000212	meiotic spindle organization
GO:0001703	gastrulation with mouth forming first
GO:0006270	DNA-dependent DNA replication initiation
GO:0006334	nucleosome assembly
GO:0006464	protein modification process
GO:0007052	mitotic spindle organization
GO:0008152	metabolic process
GO:0008406	gonad development
GO:0009790	embryo development
GO:0010171	body morphogenesis
GO:0016477	cell migration
GO:0040002	collagen and cuticulin-based cuticle development
GO:0040015	negative regulation of multicellular organism growth
GO:0040025	vulval development
GO:0040027	negative regulation of vulval development
GO:0040035	hermaphrodite genitalia development
GO:0042078	germ-line stem cell division
GO:0045132	meiotic chromosome segregation
GO:0048477	oogenesis
GO:0051301	cell division
GO:0006457	protein folding
GO:0006511	ubiquitin-dependent protein catabolic process
GO:0016311	dephosphorylation
GO:0006468	protein phosphorylation
GO:0006813	potassium ion transport
GO:0009786	regulation of asymmetric cell division
GO:0000184	nuclear-transcribed mRNA catabolic process, nonsense-mediated decay
GO:0006810	transport
GO:0006821	chloride transport
GO:0043058	regulation of backward locomotion

GO terms				
**Go Term ID**	**Description**		**Go Term ID**	**Description**
GO:0000212	meiotic spindle organization		GO:0015698	inorganic anion transport
GO:0001703	gastrulation with mouth forming first		GO:0016310	phosphorylation
GO:0006270	DNA-dependent DNA replication initiation		GO:0017145	stem cell division
GO:0006334	nucleosome assembly		GO:0019941	modification-dependent protein catabolic process
GO:0006464	protein modification process		GO:0022402	cell cycle process
GO:0007052	mitotic spindle organization		GO:0030001	metal ion transport
GO:0008152	metabolic process		GO:0031497	chromatin assembly
GO:0008406	gonad development		GO:0032502	developmental process
GO:0009790	embryo development		GO:0034728	nucleosome organization
GO:0010171	body morphogenesis		GO:0035264	multicellular organism growth
GO:0016477	cell migration		GO:0040012	regulation of locomotion
GO:0040002	collagen and cuticulin-based cuticle development		GO:0040014	regulation of multicellular organism growth
GO:0040015	negative regulation of multicellular organism growth		GO:0040028	regulation of vulval development
GO:0040025	vulval development		GO:0043057	backward locomotion
GO:0040027	negative regulation of vulval development		GO:0043412	macromolecule modification
GO:0040035	hermaphrodite genitalia development		GO:0044267	cellular protein metabolic process
GO:0042078	germ-line stem cell division		GO:0045137	development of primary sexual characteristics
GO:0045132	meiotic chromosome segregation		GO:0045926	negative regulation of growth
GO:0048477	oogenesis		GO:0048513	organ development
GO:0051301	cell division		GO:0048569	post-embryonic organ development
GO:0006457	protein folding		GO:0048608	reproductive structure development
GO:0006511	ubiquitin-dependent protein catabolic process		GO:0048610	cellular process involved in reproduction
GO:0016311	dephosphorylation		GO:0048806	genitalia development
GO:0006468	protein phosphorylation		GO:0048870	cell motility
GO:0006813	potassium ion transport		GO:0051234	establishment of localization
GO:0009786	regulation of asymmetric cell division		GO:0051241	negative regulation of multicellular organismal process
GO:0000184	nuclear-transcribed mRNA catabolic process, nonsense-mediated decay		GO:0051302	regulation of cell division
GO:0006810	transport		GO:0051321	meiotic cell cycle
GO:0006821	chloride transport		GO:0061064	negative regulation of nematode larval development
GO:0043058	regulation of backward locomotion		GO:0065004	protein-DNA complex assembly
GO:0000278	mitotic cell cycle		GO:0000226	microtubule cytoskeleton organization
GO:0000956	nuclear-transcribed mRNA catabolic process		GO:0000279	M phase
GO:0002119	nematode larval development		GO:0002164	larval development
GO:0006259	DNA metabolic process		GO:0003006	developmental process involved in reproduction
GO:0006261	DNA-dependent DNA replication		GO:0006260	DNA replication
GO:0006796	phosphate metabolic process		GO:0006323	DNA packaging

GO:0007051	spindle organization		GO:0006325	chromatin organization
GO:0007059	chromosome segregation		GO:0006333	chromatin assembly or disassembly
GO:0007126	meiosis		GO:0006402	mRNA catabolic process
GO:0007275	multicellular organismal development		GO:0006793	phosphorus metabolic process
GO:0007281	germ cell development		GO:0006812	cation transport
GO:0007292	female gamete generation		GO:0006820	anion transport
GO:0007369	gastrulation		GO:0006928	cellular component movement
GO:0007592	protein-based cuticle development		GO:0007049	cell cycle
GO:0008150	biological_process		GO:0007276	gamete generation
GO:0008356	asymmetric cell division		GO:0007548	sex differentiation
GO:0009653	anatomical structure morphogenesis		GO:0019538	protein metabolic process
GO:0009987	cellular process		GO:0022403	cell cycle phase
GO:0015672	monovalent inorganic cation transport		GO:0022414	reproductive process
GO:0032501	multicellular organismal process		GO:0051093	negative regulation of developmental process
GO:0033058	directional locomotion		GO:0051276	chromosome organization
GO:0034621	cellular macromolecular complex subunit organization		GO:0065003	macromolecular complex assembly
GO:0034622	cellular macromolecular complex assembly		GO:0065007	biological regulation
GO:0040007	growth		GO:0071103	DNA conformation change
GO:0040008	regulation of growth		GO:0071842	cellular component organization at cellular level
GO:0040011	locomotion		GO:0006996	organelle organization
GO:0042335	cuticle development		GO:0009057	macromolecule catabolic process
GO:0043170	macromolecule metabolic process		GO:0009059	macromolecule biosynthetic process
GO:0043632	modification-dependent macromolecule catabolic process		GO:0016043	cellular component organization
GO:0044260	cellular macromolecule metabolic process		GO:0016070	RNA metabolic process
GO:0048468	cell development		GO:0030163	protein catabolic process
GO:0048519	negative regulation of biological process		GO:0032504	multicellular organism reproduction
GO:0048581	negative regulation of post-embryonic development		GO:0034641	cellular nitrogen compound metabolic process
GO:0048598	embryonic morphogenesis		GO:0044085	cellular component biogenesis
GO:0048731	system development		GO:0044248	cellular catabolic process
GO:0048856	anatomical structure development		GO:0044249	cellular biosynthetic process
GO:0050789	regulation of biological process		GO:0050793	regulation of developmental process
GO:0050794	regulation of cellular process		GO:0071841	cellular component organization or biogenesis at cellular level
GO:0051179	localization		GO:2000026	regulation of multicellular organismal development
GO:0051239	regulation of multicellular organismal process		GO:0006807	nitrogen compound metabolic process
GO:0051327	M phase of meiotic cell cycle		GO:0009056	catabolic process
GO:0051603	proteolysis involved in cellular protein catabolic process		GO:0009058	biosynthetic process
GO:0051674	localization of cell		GO:0071840	cellular component organization or biogenesis
GO:0061062	regulation of nematode larval development		GO:0009791	post-embryonic development
GO:0071824	protein-DNA complex subunit organization		GO:0016071	mRNA metabolic process
GO:0071844	cellular component assembly at cellular level		GO:0019953	sexual reproduction

GO:0090304	nucleic acid metabolic process		GO:0022607	cellular component assembly
GO:0000003	reproduction		GO:0030154	cell differentiation
GO:0006139	nucleobase, nucleoside, nucleotide and nucleic acid metabolic process		GO:0034645	cellular macromolecule biosynthetic process
GO:0006401	RNA catabolic process		GO:0043933	macromolecular complex subunit organization
GO:0006508	proteolysis		GO:0044237	cellular metabolic process
GO:0006811	ion transport		GO:0044238	primary metabolic process
GO:0007010	cytoskeleton organization		GO:0044257	cellular protein catabolic process
GO:0007017	microtubule-based process		GO:0044265	cellular macromolecule catabolic process
GO:0048869	cellular developmental process		GO:0048580	regulation of post-embryonic development
GO:0048609	multicellular organismal reproductive process			
