# Supplementary material for: Gene Expression Modifications by Temperature-Toxicants Interactions in Caenorhabditis elegans
Source: PLoS One. 2011 Sep 9;6(9):e24676. doi: 10.1371/journal.pone.0024676 (PMC3170376; doi:10.1371/journal.pone.0024676)
Supplement: Figure S4 — Cellular Component GO tree representation for significantly enriched terms and their parents. Color indicated enrichment in each treatment. The figure is followed by a table with GO terms ID and description for the enriched terms and a table with all the GO terms ID and description in the figure. (PDF) [file pone.0024676.s004.pdf]

Cellular Component (GO:0005575)

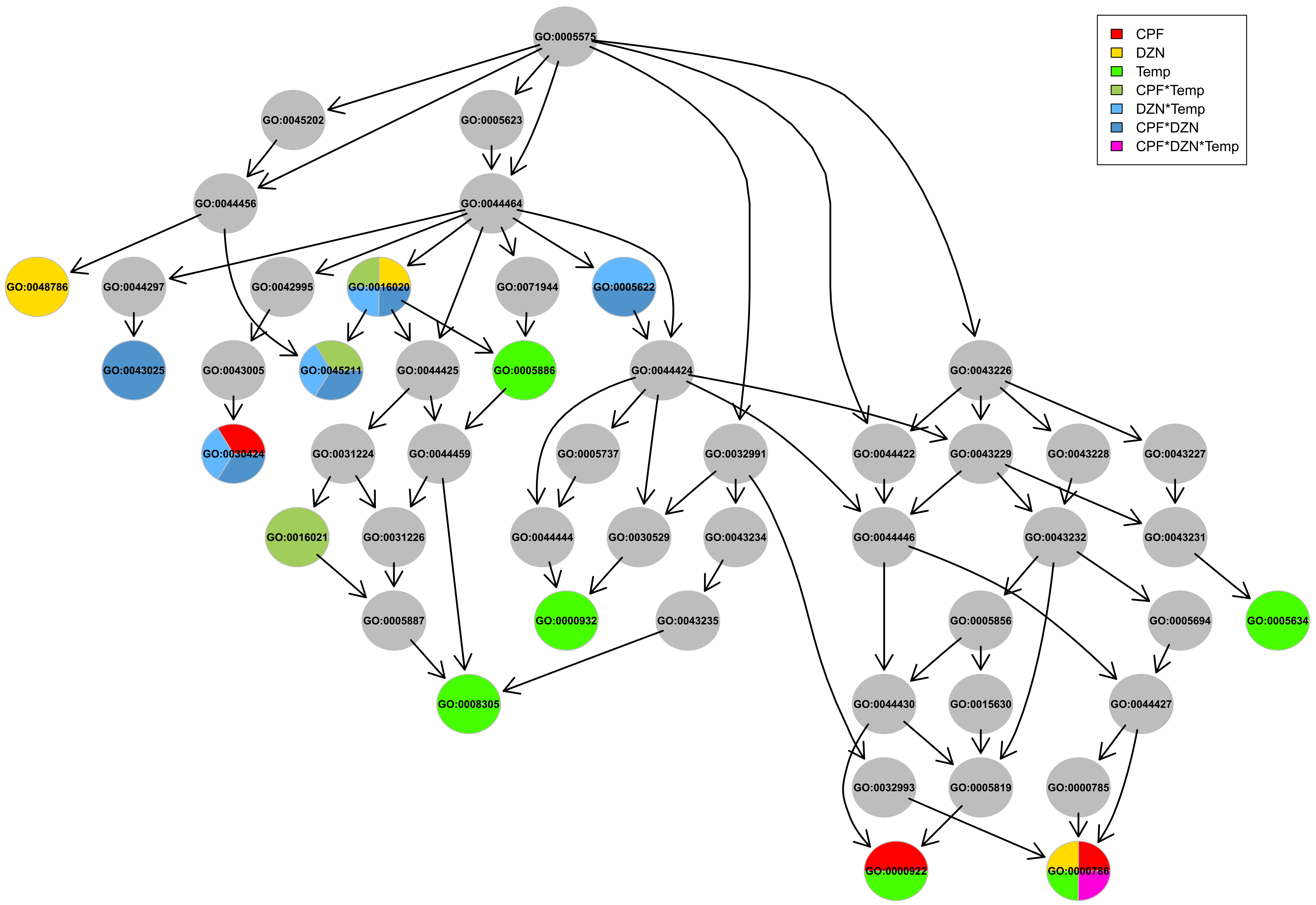

Significant GO terms in at least one treatment	
**Go Term ID**	**Description**
GO:0000786	nucleosome
GO:0000922	spindle pole
GO:0030424	axon
GO:0016020	membrane
GO:0048786	presynaptic active zone
GO:0000932	cytoplasmic mRNA processing body
GO:0005634	nucleus
GO:0005886	plasma membrane
GO:0008305	integrin complex
GO:0016021	integral to membrane
GO:0045211	postsynaptic membrane
GO:0005622	intracellular
GO:0043025	neuronal cell body

GO term ID	Description		GO term ID	Description
GO:0000786	nucleosome		GO:0044464	cell part
GO:0000922	spindle pole		GO:0071944	cell periphery
GO:0030424	axon		GO:0005575	cellular_component
GO:0016020	membrane		GO:0005623	cell
GO:0048786	presynaptic active zone		GO:0005694	chromosome
GO:0000932	cytoplasmic mRNA processing body		GO:0005737	cytoplasm
GO:0005634	nucleus		GO:0005856	cytoskeleton
GO:0005886	plasma membrane		GO:0015630	microtubule cytoskeleton
GO:0008305	integrin complex		GO:0031226	intrinsic to plasma membrane
GO:0016021	integral to membrane		GO:0032991	macromolecular complex
GO:0045211	postsynaptic membrane		GO:0042995	cell projection
GO:0005622	intracellular		GO:0043227	membrane-bounded organelle
GO:0043025	neuronal cell body		GO:0043229	intracellular organelle
GO:0000785	chromatin		GO:0043232	intracellular non-membrane-bounded organelle
GO:0005819	spindle		GO:0043234	protein complex
GO:0005887	integral to plasma membrane		GO:0044424	intracellular part
GO:0030529	ribonucleoprotein complex		GO:0044425	membrane part
GO:0031224	intrinsic to membrane		GO:0044446	intracellular organelle part
GO:0032993	protein-DNA complex		GO:0045202	synapse
GO:0043005	neuron projection		GO:0043226	organelle
GO:0043231	intracellular membrane-bounded organelle		GO:0043228	non-membrane-bounded organelle
GO:0043235	receptor complex		GO:0044422	organelle part
GO:0044297	cell body			
GO:0044427	chromosomal part			
GO:0044430	cytoskeletal part			
GO:0044444	cytoplasmic part			
GO:0044456	synapse part			
GO:0044459	plasma membrane part			
