# Supplementary material for: Gene Expression Modifications by Temperature-Toxicants Interactions in Caenorhabditis elegans
Source: PLoS One. 2011 Sep 9;6(9):e24676. doi: 10.1371/journal.pone.0024676 (PMC3170376; doi:10.1371/journal.pone.0024676)
Supplement: Table S2 — List of commonly regulated genes by all toxicants treatments at each temperature: 10 genes at 16°C and 53 at 24°C ( Figure 1 ). (DOC) [file pone.0024676.s006.doc]

| **Sequence name** | **Gene name** | **CPF p-value**  **16˚C** | **DZN p-value 16˚C** | **CPF+DZN p-value 16˚C** |
| --- | --- | --- | --- | --- |
| K09D9.2 | cyp-35A3 | 0.000308 | 0.00629 | 5.40E-05 |
| Y19D10A.9 | clec-209 | 0.003771 | 0.000677 | 1.80E-05 |
| W02G9.4 | W02G9.4 | 0.002478 | 0.001342 | 0.004728 |
| F59A7.1 | clec-206 | 0.005479 | 0.008021 | 0.002018 |
| C06B3.3 | cyp-35C1 | 0.000788 | 8.30E-05 | 0.000256 |
| ZK678.5 | wrt-4 | 0.000135 | 0.004877 | 0.007776 |
| C15C8.3 | C15C8.3 | 0.001522 | 0.000337 | 0.002632 |
| F25D1.5 | F25D1.5 | 0.002481 | 0.000318 | 0.003724 |
| T16G1.6 | T16G1.6 | 1.20E-05 | 0.007901 | 0.000371 |
| R06A10.2 | gsa-1 | 0.008204 | 0.007894 | 0.000109 |

| **Sequence name** | **Gene name** | **CPF p-value 24˚C** | **DZN p-value 24˚C** | **CPF+DZN p-value 24˚C** |
| --- | --- | --- | --- | --- |
| B0213.6 | nlp-31 | 0.006883 | 0.002993 | 0.006262 |
| C02A12.4 | lys-7 | 0.000106 | 0.003652 | 1.60E-05 |
| C06B3.3 | cyp-35C1 | 0.001447 | 0.00223 | 0.000367 |
| C15C8.3 | C15C8.3 | 0.00272 | 0.009786 | 0.002299 |
| C17B7.2 | C17B7.2 | 0.000956 | 0.000759 | 0.006146 |
| C27H5.4 | C27H5.4 | 0.005744 | 0.000277 | 0.003362 |
| C29F7.1 | C29F7.1 | 0.004018 | 0.002527 | 0.000346 |
| C29F7.2 | C29F7.2 | 1.50E-05 | 0.000691 | 5.00E-06 |
| C49G7.8 | cyp-35A4 | 0.000699 | 0.002563 | 3.20E-05 |
| C54D1.2 | clec-86 | 0.002153 | 0.006449 | 0.000226 |
| F01D4.2 | ugt-44 | 0.00545 | 0.003202 | 0.007709 |
| F01D5.9 | cyp-37A1 | 0.00034 | 0.000186 | 3.50E-05 |
| F08G5.6 | F08G5.6 | 2.00E-06 | 0.000999 | 7.90E-05 |
| F13D11.4 | F13D11.4 | 0.001471 | 0.004539 | 0.000112 |
| F13D12.7 | gpb-1 | 0.00614 | 0.008421 | 0.006427 |
| F13H6.4 | F13H6.4 | 0.003209 | 0.002186 | 0.000668 |
| F16B4.9 | nhr-178 | 0.006596 | 0.004689 | 0.008388 |
| F25D1.5 | F25D1.5 | 0.001194 | 0.003033 | 9.00E-06 |
| F37H8.4 | sfxn-1.2 | 0.009412 | 0.004332 | 9.20E-05 |
| F38A5.2 | F38A5.2 | 0.009618 | 0.005524 | 0.007462 |
| F41H10.3 | F41H10.3 | 0.000461 | 0.000112 | 0.007764 |
| F42A6.7 | hrp-1 | 0.008689 | 0.002662 | 0.0083 |
| F48G7.8 | F48G7.8 | 0.004461 | 0.003738 | 0.002668 |
| F49C12.7 | F49C12.7 | 0.000127 | 0.000118 | 1.10E-05 |
| F52H3.2 | F52H3.2 | 0.003124 | 0.004954 | 0.002246 |
| F53C11.3 | F53C11.3 | 0.005412 | 0.003217 | 0.001885 |
| F55A12.4 | dhs-2 | 0.000157 | 1.50E-05 | 0.000199 |
| F59A7.1 | clec-206 | 0.000305 | 0.001653 | 1.10E-05 |
| F59D8.1 | vit-3 | 0.003083 | 0.000208 | 0.004524 |
| F59D8.2 | vit-4 | 0.002635 | 0.001066 | 0.000183 |
| H25K10.4 | H25K10.4 | 0.001625 | 0.003636 | 0.00034 |
| K01D12.12 | cdr-6 | 0.000142 | 0.000979 | 1.20E-05 |
| K11E8.1 | unc-43 | 0.008269 | 3.50E-05 | 0.005071 |
| M02A10.3 | sli-1 | 0.000881 | 0.002552 | 0.002263 |
| R02E12.6 | hrg-1 | 0.001208 | 0.002261 | 3.10E-05 |
| T06C12.10 | cgt-1 | 0.007396 | 0.000928 | 0.00104 |
| T08B1.3 | alh-5 | 0.009072 | 0.000522 | 0.000834 |
| T08D2.3 | T08D2.3 | 0.002167 | 0.000729 | 0.00145 |
| T10H4.12 | cpr-3 | 0.002462 | 0.003116 | 0.000552 |
| T11F9.11 | dhs-19 | 0.004049 | 0.000314 | 0.006538 |
| T16G1.6 | T16G1.6 | 0.000202 | 0.000149 | 7.20E-05 |
| T16G1.7 | T16G1.7 | 9.10E-05 | 0.005932 | 0.000552 |
| T17E9.2 | nmt-1 | 0.004319 | 0.003525 | 0.000886 |
| T20B3.14 | T20B3.14 | 0.005419 | 0.0019 | 0.001355 |
| T28A11.19 | T28A11.19 | 0.006099 | 0.004058 | 0.000506 |
| T28D9.3 | T28D9.3 | 0.007787 | 0.00016 | 6.80E-05 |
| W01A11.1 | W01A11.1 | 0.000158 | 1.70E-05 | 2.60E-05 |
| W09H1.6 | lec-1 | 0.007303 | 0.004712 | 0.000102 |
| Y14H12B.1 | Y14H12B.1 | 0.005195 | 1.40E-05 | 0.003279 |
| Y40H7A.10 | Y40H7A.10 | 0.009741 | 0.000405 | 0.001438 |
| Y46C8AL.2 | clec-174 | 0.000977 | 0.002146 | 0.000267 |
| Y56A3A.29 | ung-1 | 0.002867 | 0.006186 | 0.001928 |
| Y73F8A.25 | Y73F8A.25 | 0.008826 | 4.00E-06 | 0.001949 |
