# Supplementary material for: Gene Expression Modifications by Temperature-Toxicants Interactions in Caenorhabditis elegans
Source: PLoS One. 2011 Sep 9;6(9):e24676. doi: 10.1371/journal.pone.0024676 (PMC3170376; doi:10.1371/journal.pone.0024676)
Supplement: Table S5 — List of commonly regulated genes per toxicant at both temperatures: 75 genes for CPF, 72 genes for DZN, and 112 genes for CPF+DZN ( Figure 3 ). (DOC) [file pone.0024676.s009.doc]

| **Sequence name** | **Gene name** | **CPF p-value 16˚C** | **CPF p-value 24˚C** |
| --- | --- | --- | --- |
| T24H10.2 | - | 0.002276 | 0.004467 |
| Y51A2B.1 | - | 0.008773 | 0.000911 |
| C55C2.5 | aat-5 | 0.004316 | 0.007081 |
| T08B1.3 | alh-5 | 0.000273 | 0.009072 |
| F45E4.1 | arf-1.1 | 0.00979 | 0.004713 |
| C15C8.3 | C15C8.3 | 0.001522 | 0.00272 |
| C17B7.4 | C17B7.4 | 0.003516 | 0.008758 |
| C24A1.2 | C24A1.2 | 0.004924 | 0.003078 |
| C29F7.1 | C29F7.1 | 0.001944 | 0.004018 |
| C30G12.2 | C30G12.2 | 0.008756 | 0.003965 |
| K01D12.12 | cdr-6 | 0.0031 | 0.000142 |
| T06C12.10 | cgt-1 | 0.000332 | 0.007396 |
| F59A7.1 | clec-206 | 0.005479 | 0.000305 |
| C14A6.1 | clec-48 | 0.004221 | 0.006556 |
| Y46C8AL.5 | clec-72 | 0.007011 | 0.001855 |
| C54D1.2 | clec-86 | 0.002295 | 0.002153 |
| F42A6.4 | cyp-25A5 | 0.002538 | 0.008449 |
| T19B10.1 | cyp-29A2 | 0.002072 | 0.000158 |
| C49G7.8 | cyp-35A4 | 0.000175 | 0.000699 |
| C06B3.3 | cyp-35C1 | 0.000788 | 0.001447 |
| F01D5.9 | cyp-37A1 | 0.005278 | 0.00034 |
| ZC373.6 | dao-4 | 0.002659 | 0.008956 |
| T11F9.11 | dhs-19 | 0.000209 | 0.004049 |
| F55A12.4 | dhs-2 | 9.10E-05 | 0.000157 |
| F35B12.2 | dhs-20 | 0.001586 | 0.001288 |
| F08G5.6 | F08G5.6 | 0.000993 | 2.00E-06 |
| F10D7.5 | F10D7.5 | 0.001476 | 0.000112 |
| F13D11.4 | F13D11.4 | 0.00397 | 0.001471 |
| F15E11.12 | F15E11.12 | 0.008715 | 0.004387 |
| F18E3.7 | F18E3.7 | 0.004732 | 0.007514 |
| F25D1.5 | F25D1.5 | 0.002481 | 0.001194 |
| F35E2.3 | F35E2.3 | 0.006182 | 0.002674 |
| F38C2.1 | F38C2.1 | 0.005581 | 0.000134 |
| F43G9.2 | F43G9.2 | 0.007747 | 0.000298 |
| F44D12.4 | F44D12.4 | 0.008729 | 0.003531 |
| F49C12.7 | F49C12.7 | 0.000168 | 0.000127 |
| F49F1.5 | F49F1.5 | 0.001118 | 0.004176 |
| F58E6.1 | F58E6.1 | 0.003621 | 0.003527 |
| F33D4.3 | flp-13 | 0.004714 | 0.007988 |
| F31F6.4 | flp-8 | 0.005351 | 0.009298 |
| Y48E1B.10 | gst-20 | 0.003415 | 0.00506 |
| R03D7.6 | gst-5 | 0.003541 | 0.001153 |
| F11G11.2 | gst-7 | 0.005318 | 0.009856 |
| Y73B6BL.9 | hil-2 | 0.00497 | 0.002079 |
| E03A3.4 | his-70 | 0.009202 | 0.003287 |
| K04G2.7 | K04G2.7 | 0.003284 | 0.006229 |
| M02D8.4 | M02D8.4 | 0.003305 | 0.004747 |
| M03F8.1 | M03F8.1 | 0.009395 | 0.003963 |
| C04G6.1 | mpk-2 | 0.004947 | 0.006833 |
| K05F1.7 | msp-63 | 0.006828 | 0.006807 |
| R04B5.3 | nhr-205 | 9.30E-05 | 0.004726 |
| T27B1.1 | osm-1 | 0.001367 | 0.00237 |
| F58A6.10 | srb-12 | 0.006017 | 0.001002 |
| T28H11.6 | ssp-11 | 0.000608 | 0.002618 |
| T08D2.3 | T08D2.3 | 0.000128 | 0.002167 |
| T10B5.8 | T10B5.8 | 0.002883 | 0.008411 |
| T16G1.6 | T16G1.6 | 1.20E-05 | 0.000202 |
| T22D1.11 | T22D1.11 | 0.000794 | 0.001305 |
| H23N18.1 | ugt-13 | 0.002111 | 0.002776 |
| F01D4.2 | ugt-44 | 0.000421 | 0.00545 |
| T19H12.1 | ugt-9 | 0.007303 | 0.006212 |
| K07E3.8 | vem-1 | 3.00E-05 | 0.000508 |
| K09F5.2 | vit-1 | 0.006825 | 0.005793 |
| F59D8.1 | vit-3 | 0.007863 | 0.003083 |
| F59D8.2 | vit-4 | 0.002201 | 0.002635 |
| W01A11.1 | W01A11.1 | 5.00E-06 | 0.000158 |
| ZK678.5 | wrt-4 | 0.000135 | 0.001513 |
| Y32F6A.5 | Y32F6A.5 | 0.009484 | 0.000808 |
| Y37H2A.11 | Y37H2A.11 | 0.006321 | 0.000272 |
| Y41C4A.11 | Y41C4A.11 | 0.007863 | 0.001429 |
| Y53H1B.2 | Y53H1B.2 | 0.001598 | 0.001529 |
| Y57A10A.15 | Y57A10A.15 | 0.000162 | 0.009113 |
| Y67A6A.1 | Y67A6A.1 | 0.003999 | 0.004298 |
| Y69A2AR.32 | Y69A2AR.32 | 0.007623 | 0.000624 |
| Y9D1A.2 | Y9D1A.2 | 0.0015 | 0.009596 |

| **Sequence name** | **Gene name** | **DZN p-value 16˚C** | **DZN p-value 24˚C** |
| --- | --- | --- | --- |
| M04C9.6 | - | 0.00409 | 0.006782 |
| Y73B6BL.15 | - | 0.005453 | 0.002881 |
| F54H12.1 | aco-2 | 0.005558 | 0.003329 |
| D1022.7 | aka-1 | 0.003575 | 0.000573 |
| T08B1.3 | alh-5 | 0.005407 | 0.000522 |
| B0238.13 | B0238.13 | 0.00461 | 0.003916 |
| T07E3.5 | brc-2 | 0.008285 | 0.00042 |
| C01B10.10 | C01B10.10 | 0.009859 | 0.004803 |
| C04E6.5 | C04E6.5 | 0.000747 | 0.00603 |
| C15C8.3 | C15C8.3 | 0.000337 | 0.009786 |
| C18G1.1 | C18G1.1 | 0.008123 | 0.001547 |
| C18H9.6 | C18H9.6 | 0.000629 | 0.009123 |
| C29F7.2 | C29F7.2 | 0.000297 | 0.000691 |
| C48B4.11 | C48B4.11 | 0.009933 | 2.40E-05 |
| C50B6.9 | C50B6.9 | 0.004573 | 0.003908 |
| C52A10.2 | C52A10.2 | 0.000954 | 0.002754 |
| C55C3.6 | C55C3.6 | 0.000882 | 0.004838 |
| C03H5.1 | clec-10 | 0.001488 | 0.003598 |
| F59A7.1 | clec-206 | 0.008021 | 0.001653 |
| Y19D10A.9 | clec-209 | 0.000677 | 0.00054 |
| C54D1.2 | clec-86 | 0.003201 | 0.006449 |
| Y23H5A.5 | ctn-1 | 0.001274 | 0.000527 |
| F42A6.4 | cyp-25A5 | 0.000678 | 0.000884 |
| B0213.16 | cyp-34A10 | 1.10E-05 | 0.000536 |
| K09D9.2 | cyp-35A3 | 0.00629 | 0.001409 |
| C06B3.3 | cyp-35C1 | 8.30E-05 | 0.00223 |
| T05C12.5 | dylt-3 | 0.008818 | 0.0046 |
| T07A9.5 | eri-1 | 0.007295 | 0.002069 |
| F10G7.10 | F10G7.10 | 0.008039 | 0.000842 |
| F13A7.7 | F13A7.7 | 0.007185 | 0.005325 |
| F25D1.5 | F25D1.5 | 0.000318 | 0.003033 |
| F28H1.5 | F28H1.5 | 0.002246 | 0.006371 |
| F42A10.3 | F42A10.3 | 0.000628 | 0.007044 |
| F47B3.7 | F47B3.7 | 0.005957 | 0.00054 |
| F49F1.6 | F49F1.6 | 0.008021 | 0.003212 |
| H38K22.5 | gly-6 | 5.50E-05 | 0.004293 |
| H06O01.4 | H06O01.4 | 0.004547 | 0.002245 |
| H23N18.4 | H23N18.4 | 0.005565 | 0.008159 |
| H25K10.4 | H25K10.4 | 0.004279 | 0.003636 |
| T09E8.2 | him-17 | 0.003832 | 0.004382 |
| Y73B6BL.2 | htp-2 | 0.000828 | 0.002487 |
| M7.2 | klc-1 | 0.004789 | 0.00776 |
| M01G5.3 | M01G5.3 | 0.005865 | 0.000281 |
| Y69A2AR.30 | mdf-2 | 0.008803 | 0.005405 |
| C38D4.3 | mel-28 | 0.005612 | 0.003308 |
| T23B7.1 | nspd-4 | 0.001817 | 0.008232 |
| F08F8.1 | numr-2 | 0.000909 | 0.004214 |
| ZK455.7 | pgp-3 | 0.002873 | 0.000815 |
| D2030.6 | prg-1 | 0.002203 | 0.002907 |
| F16H9.1 | rgs-2 | 0.001337 | 0.008372 |
| Y57A10A.19 | rsr-2 | 0.002013 | 0.001937 |
| M02A10.3 | sli-1 | 0.008105 | 0.002552 |
| F08B12.3 | slo-2 | 0.007709 | 0.001171 |
| AC7.2 | soc-2 | 0.002553 | 0.008088 |
| K11D12.3 | srr-4 | 0.00488 | 0.001997 |
| C07E3.1 | stip-1 | 0.002819 | 0.001617 |
| T09E8.1 | T09E8.1 | 0.007811 | 0.00535 |
| T09F5.10 | T09F5.10 | 3.50E-05 | 0.004634 |
| T16G1.6 | T16G1.6 | 0.007901 | 0.000149 |
| T20D4.11 | T20D4.11 | 0.009972 | 0.000317 |
| T25B9.6 | T25B9.6 | 0.00478 | 0.001213 |
| ZC581.6 | try-7 | 0.004884 | 0.005231 |
| F25B5.4 | ubq-1 | 0.002588 | 0.003084 |
| H23N18.3 | ugt-8 | 0.002961 | 0.005198 |
| C50C3.9 | unc-36 | 0.002388 | 0.000323 |
| W02G9.4 | W02G9.4 | 0.001342 | 0.005173 |
| Y40H7A.10 | Y40H7A.10 | 0.009135 | 0.000405 |
| Y41E3.5 | Y41E3.5 | 0.00885 | 0.009981 |
| Y43C5B.3 | Y43C5B.3 | 0.008876 | 0.003561 |
| Y47H10A.5 | Y47H10A.5 | 0.006823 | 0.000646 |
| Y53F4B.14 | Y53F4B.14 | 0.009185 | 0.002356 |
| Y58G8A.4 | Y58G8A.4 | 0.000446 | 0.007056 |

| **Sequence name** | **Gene name** | **CPF+DZN p-value 16˚C** | **CPF+DZN p-value 24˚C** |
| --- | --- | --- | --- |
| T06E8.1 | acl-2 | 0.002074 | 0.000848 |
| T11B7.4 | alp-1 | 0.00789 | 0.005924 |
| B0244.9 | B0244.9 | 0.006092 | 0.007729 |
| C05D2.4 | bas-1 | 0.002058 | 0.008017 |
| C07G3.2 | C07G3.2 | 0.007502 | 0.002321 |
| C14C6.5 | C14C6.5 | 0.000765 | 0.000912 |
| C15C8.3 | C15C8.3 | 0.002632 | 0.002299 |
| C17B7.4 | C17B7.4 | 0.004329 | 0.002292 |
| C18H9.6 | C18H9.6 | 0.000358 | 0.00015 |
| C24H12.4 | C24H12.4 | 0.00605 | 0.003568 |
| C29F3.7 | C29F3.7 | 0.000888 | 0.006194 |
| C29F7.2 | C29F7.2 | 0.00026 | 5.00E-06 |
| C30G12.2 | C30G12.2 | 0.000871 | 0.001297 |
| C32H11.4 | C32H11.4 | 0.008509 | 0.006786 |
| C34C12.5 | C34C12.5 | 0.007436 | 0.004287 |
| C38C10.2 | C38C10.2 | 0.009219 | 0.005745 |
| C47E12.10 | C47E12.10 | 0.007376 | 0.001545 |
| C49H3.6 | C49H3.6 | 0.000927 | 0.000238 |
| C52A10.1 | C52A10.1 | 0.002297 | 0.000322 |
| C52A10.2 | C52A10.2 | 0.006201 | 5.00E-06 |
| C53A3.2 | C53A3.2 | 0.001513 | 0.000516 |
| B0511.9 | cdc-26 | 0.006226 | 0.001171 |
| F38A1.5 | clec-166 | 0.00354 | 0.000836 |
| Y46C8AL.2 | clec-174 | 0.00052 | 0.000267 |
| F59A7.1 | clec-206 | 0.002018 | 1.10E-05 |
| Y19D10A.9 | clec-209 | 1.80E-05 | 5.50E-05 |
| C35D10.14 | clec-5 | 0.008182 | 0.005144 |
| F35C5.9 | clec-66 | 0.005942 | 0.003264 |
| Y46C8AL.5 | clec-72 | 0.000577 | 4.20E-05 |
| ZK652.9 | coq-5 | 0.009482 | 0.004237 |
| T10H4.12 | cpr-3 | 0.000852 | 0.000552 |
| B0024.14 | crm-1 | 0.005354 | 0.000919 |
| C36A4.2 | cyp-25A2 | 0.008886 | 0.000133 |
| B0213.16 | cyp-34A10 | 0.001136 | 0.000531 |
| C03G6.15 | cyp-35A2 | 0.00223 | 4.70E-05 |
| K09D9.2 | cyp-35A3 | 5.40E-05 | 0.003175 |
| C49G7.8 | cyp-35A4 | 1.40E-05 | 3.20E-05 |
| K07C6.5 | cyp-35A5 | 0.001497 | 0.000888 |
| C06B3.3 | cyp-35C1 | 0.000256 | 0.000367 |
| B0432.2 | djr-1.1 | 0.000333 | 0.002335 |
| C31H2.2 | dpy-8 | 0.004754 | 0.009854 |
| E02D9.1 | E02D9.1 | 0.007748 | 0.003923 |
| F56H11.3 | elo-7 | 0.001362 | 0.009811 |
| K10D2.6 | emb-8 | 0.002269 | 0.002727 |
| F07A5.2 | F07A5.2 | 0.005455 | 0.008628 |
| F08G5.6 | F08G5.6 | 0.000591 | 7.90E-05 |
| F13D12.6 | F13D12.6 | 0.003096 | 0.003067 |
| F13H6.3 | F13H6.3 | 0.001726 | 0.000244 |
| F13H6.4 | F13H6.4 | 0.005373 | 0.000668 |
| F15E11.12 | F15E11.12 | 0.00052 | 0.000188 |
| F22B8.6 | F22B8.6 | 0.007166 | 0.00281 |
| F25D1.5 | F25D1.5 | 0.003724 | 9.00E-06 |
| F35E12.5 | F35E12.5 | 0.009126 | 0.006938 |
| F38C2.1 | F38C2.1 | 0.004895 | 0.000259 |
| F40A3.5 | F40A3.5 | 0.000558 | 0.004574 |
| F42A10.7 | F42A10.7 | 0.002343 | 0.009092 |
| F43E2.6 | F43E2.6 | 0.004471 | 0.002337 |
| F44E7.2 | F44E7.2 | 0.005821 | 0.001657 |
| F48G7.12 | F48G7.12 | 0.000626 | 0.009195 |
| F49C12.7 | F49C12.7 | 0.001038 | 1.10E-05 |
| F49F1.7 | F49F1.7 | 0.006825 | 0.001692 |
| F55G11.2 | F55G11.2 | 0.00555 | 0.003374 |
| F55G11.3 | F55G11.3 | 0.000769 | 0.000987 |
| F55G11.4 | F55G11.4 | 0.001937 | 1.10E-05 |
| F59B1.8 | F59B1.8 | 0.001253 | 0.009508 |
| B0391.9 | fbxa-139 | 0.005961 | 0.000954 |
| H24K24.5 | fmo-5 | 0.002275 | 0.000554 |
| K10B3.7 | gpd-3 | 0.003716 | 0.003505 |
| F11G11.2 | gst-7 | 0.00069 | 0.002552 |
| H19M22.3 | H19M22.3 | 0.007946 | 0.009514 |
| H25K10.4 | H25K10.4 | 0.006846 | 0.00034 |
| F20B6.8 | hpk-1 | 0.005565 | 0.002914 |
| K04A8.10 | K04A8.10 | 0.008421 | 0.000436 |
| K04C1.2 | K04C1.2 | 0.006623 | 0.001087 |
| K08D12.6 | K08D12.6 | 0.000386 | 0.003143 |
| K12H4.7 | K12H4.7 | 0.007611 | 0.002442 |
| M7.2 | klc-1 | 0.001107 | 0.003364 |
| T02G5.9 | krs-1 | 0.001603 | 0.001389 |
| M28.6 | lact-3 | 0.008141 | 0.000105 |
| W01A11.4 | lec-10 | 0.007455 | 1.50E-05 |
| T24A11.1 | mtm-3 | 7.30E-05 | 0.002351 |
| EEED8.8 | ndx-6 | 0.000574 | 0.006555 |
| F16B4.9 | nhr-178 | 0.007232 | 0.008388 |
| F36G9.12 | oac-20 | 0.008388 | 0.00313 |
| ZK455.7 | pgp-3 | 0.002934 | 0.000563 |
| Y80D3A.7 | ptr-22 | 0.001337 | 0.006261 |
| R102.2 | R102.2 | 0.00409 | 0.000342 |
| W06A7.3 | ret-1 | 0.005088 | 0.001516 |
| T16G1.6 | T16G1.6 | 0.000371 | 7.20E-05 |
| T21H3.1 | T21H3.1 | 0.003172 | 0.000232 |
| T28A11.5 | T28A11.5 | 0.00802 | 0.001074 |
| C02F12.1 | tsp-17 | 0.009954 | 0.004844 |
| T28B4.3 | ttr-6 | 0.000987 | 0.000444 |
| T19H12.11 | ugt-10 | 0.004238 | 0.006338 |
| ZC455.6 | ugt-5 | 0.006135 | 0.00016 |
| C03A7.11 | ugt-51 | 0.008659 | 0.004856 |
| H23N18.3 | ugt-8 | 0.002988 | 0.005384 |
| W01A11.3 | unc-83 | 0.00926 | 0.004271 |
| W01A11.1 | W01A11.1 | 0.004835 | 2.60E-05 |
| W02G9.4 | W02G9.4 | 0.004728 | 0.001546 |
| W04B5.3 | W04B5.3 | 0.004415 | 0.009498 |
| W06F12.2 | W06F12.2 | 0.005838 | 0.008838 |
| Y32F6A.4 | Y32F6A.4 | 0.00248 | 0.004825 |
| Y32F6A.5 | Y32F6A.5 | 0.001589 | 0.003606 |
| Y38A10A.7 | Y38A10A.7 | 0.007713 | 0.000854 |
| Y39B6A.1 | Y39B6A.1 | 0.002413 | 0.004683 |
| Y39B6A.24 | Y39B6A.24 | 0.005159 | 0.007556 |
| Y48G8AL.13 | Y48G8AL.13 | 0.004174 | 0.003026 |
| Y54G2A.29 | Y54G2A.29 | 0.009925 | 0.004692 |
| Y57A10A.6 | Y57A10A.6 | 0.001874 | 0.003457 |
| Y62E10A.13 | Y62E10A.13 | 0.001923 | 0.000548 |
| ZK470.2 | ZK470.2 | 0.002461 | 0.000137 |
