# Supplementary material for: Gene Expression Modifications by Temperature-Toxicants Interactions in Caenorhabditis elegans
Source: PLoS One. 2011 Sep 9;6(9):e24676. doi: 10.1371/journal.pone.0024676 (PMC3170376; doi:10.1371/journal.pone.0024676)
Supplement: Table S6 — List of enriched functional domains in regulated genes by toxicants at different temperatures (from Figure 3 and Table S5). (DOC) [file pone.0024676.s010.doc]

| **Total Genes in Domain** | **Domain ID** | **Significant Genes** | **P-value** | **Description** |
| --- | --- | --- | --- | --- |
| **CPF** | | | | |
| 78 | IPR002401 | 5 | 1.97E-07 | Cytochrome P450, E-class, group I |
| 80 | IPR001128 | 5 | 2.30E-07 | Cytochrome P450 |
| 82 | IPR002347 | 5 | 2.66E-07 | Glucose/ribitol dehydrogenase |
| 95 | IPR002198 | 5 | 6.39E-07 | Short-chain dehydrogenase/reductase SDR |
| 72 | IPR002403 | 4 | 3.62E-06 | Cytochrome P450, E-class, group IV |
| **DZN** | | | | |
| 78 | IPR002401 | 4 | 4.40E-06 | Cytochrome P450, E-class, group I |
| 80 | IPR001128 | 4 | 4.98E-06 | Cytochrome P450 |
| **CPF+DZN** | | | | |
| 49 | IPR003366 | 6 | 3.64E-09 | CUB-like region |
| 78 | IPR002401 | 7 | 4.01E-09 | Cytochrome P450, E-class, group I |
| 80 | IPR001128 | 7 | 4.92E-09 | Cytochrome P450 |
| 72 | IPR002403 | 6 | 5.70E-08 | Cytochrome P450, E-class, group IV |
| 45 | IPR002018 | 4 | 2.49E-06 | Carboxylesterase, type B |
| 87 | IPR002213 | 5 | 4.03E-06 | UDP-glucuronosyl/UDP-glucosyltransferase |
