# Supplementary material for: Gene Expression Modifications by Temperature-Toxicants Interactions in Caenorhabditis elegans
Source: PLoS One. 2011 Sep 9;6(9):e24676. doi: 10.1371/journal.pone.0024676 (PMC3170376; doi:10.1371/journal.pone.0024676)
Supplement: Table S7 — List of significantly regulated cytochrome P450 genes by CPF, DZN and temperature. (DOC) [file pone.0024676.s011.doc]

**Supplementary Table 7: Significantly affected CYP genes.**

| **Table 7A: Significantly affected CYP genes in a interaction effect model.** | | | | | | | | | | | | | | | | |
| --- | --- | --- | --- | --- | --- | --- | --- | --- | --- | --- | --- | --- | --- | --- | --- | --- |
| **Array ID** | | **Sequence name** | **Gene name** | | **CPF 16C** | | **DZN 16C** | | **CPF+DZN 16C** | | **CPF 24C** | | **DZN 24C** | | **CPF+DZN 24C** | |
| 16861 | | ZK1320.4 | cyp-13A10 | | - | | 6.62E-03 | | - | | - | | - | | - | |
| 4753 | | T10B9.2 | cyp-13A5 | | - | | 4.62E-03 | | - | | - | | - | | - | |
| 6689 | | T10B9.3 | cyp-13A6 | | - | | - | | - | | - | | 6.73E-03 | | 1.74E-04 | |
| 8997 | | K09A11.3 | cyp-14A2 | | - | | - | | - | | - | | - | | 3.75E-03 | |
| 10005 | | F08F3.7 | cyp-14A5 | | - | | - | | - | | 4.70E-04 | | - | | - | |
| 14001 | | C36A4.1 | cyp-25A1 | | - | | - | | 1.90E-03 | | - | | - | | - | |
| 16382 | | C36A4.2 | cyp-25A2 | | 6.15E-04 | | - | | 8.89E-03 | | - | | 1.45E-03 | | 1.33E-04 | |
| 2048 | | F42A6.4 | cyp-25A5 | | 2.54E-03 | | 6.78E-04 | | - | | 8.45E-03 | | 8.84E-04 | | - | |
| 2235 | | K06B9.1 | cyp-25A6 | | - | | - | | 6.50E-03 | | - | | 3.91E-04 | | - | |
| 11472 | | T19B10.1 | cyp-29A2 | | 2.07E-03 | | 6.20E-03 | | - | | 1.58E-04 | | - | | 1.64E-05 | |
| 14784 | | Y38C9B.1 | cyp-29A3 | | 4.38E-03 | | - | | - | | - | | - | | 6.38E-04 | |
| 10767 | | H02I12.8 | cyp-31A2 | | - | | - | | - | | - | | 5.62E-03 | | - | |
| 12703 | | H02I12.8 | cyp-31A2 | | - | | - | | - | | - | | - | | - | |
| 15142 | | Y17G9B.3 | cyp-31A3 | | - | | - | | - | | - | | - | | 4.90E-03 | |
| 15346 | | Y5H2B.5 | cyp-32B1 | | - | | - | | - | | - | | - | | 1.73E-03 | |
| 9449 | | R08F11.3 | cyp-33C8 | | - | | - | | - | | 1.39E-03 | | - | | - | |
| 10396 | | C50H11.15 | cyp-33C9 | | - | | 9.65E-03 | | - | | - | | - | | 1.62E-04 | |
| 18615 | | B0213.16 | cyp-34A10 | | - | | 1.14E-05 | | 1.14E-03 | | - | | 5.36E-04 | | 5.31E-04 | |
| 1774 | | B0213.15 | cyp-34A9 | | 7.51E-03 | | - | | - | | - | | 3.19E-03 | | 2.68E-05 | |
| 3614 | | C03G6.15 | cyp-35A2 | | - | | - | | 2.23E-03 | | - | | 1.44E-03 | | 4.73E-05 | |
| 250 | | K09D9.2 | cyp-35A3 | | 3.08E-04 | | 6.29E-03 | | 5.38E-05 | | - | | 1.41E-03 | | 3.17E-03 | |
| 20559 | | C49G7.8 | cyp-35A4 | | 1.75E-04 | | - | | 1.44E-05 | | 6.99E-04 | | 2.56E-03 | | 3.15E-05 | |
| 15822 | | K07C6.5 | cyp-35A5 | | - | | 7.97E-03 | | 1.50E-03 | | - | | - | | 8.88E-04 | |
| 6542 | | K07C6.4 | cyp-35B1 | | - | | - | | - | | - | | 8.82E-03 | | 9.02E-05 | |
| 12422 | | C06B3.3 | cyp-35C1 | | 7.88E-04 | | 8.32E-05 | | 2.56E-04 | | 1.45E-03 | | 2.23E-03 | | 3.67E-04 | |
| 4743 | | F01D5.9 | cyp-37A1 | | 5.28E-03 | | - | | - | | 3.40E-04 | | 1.86E-04 | | 3.54E-05 | |
| 3407 | | ZK177.5 | cyp-44A1 | | - | | - | | - | | - | | - | | 3.63E-03 | |
| **Table 7B: Significantly affected CYP genes in a single effect model.** | | | | | | | | | | | | | | | | |
| **Array ID** | **Sequence name** | | | **Gene name** | | **CPF** | **DZN** | **Temp** | | **CPF*DZN** | | **CPF*Tem** | | **DZN*Temp** | | **CPF*DZN*Temp** |
| 250 | K09D9.2 | | | cyp-35A3 | | - | 0.000161 | - | | - | | - | | - | | - |
| 352 | E03E2.1 | | | cyp-43A1 | | - | - | - | | - | | 0.007421 | | 0.009139 | | - |
| 1774 | B0213.15 | | | cyp-34A9 | | 0.004475 | - | 4.80E-05 | | - | | - | | - | | - |
| 2235 | K06B9.1 | | | cyp-25A6 | | 0.009285 | 0.000518 | - | | - | | - | | - | | - |
| 2670 | K07C6.2 | | | cyp-35B3 | | - | - | - | | - | | - | | 0.006327 | | - |
| 3407 | ZK177.5 | | | cyp-44A1 | | - | - | - | | 0.000507 | | 4.32E-04 | | - | | - |
| 3614 | C03G6.15 | | | cyp-35A2 | | - | - | - | | - | | 0.001359 | | 0.008289 | | - |
| 4421 | F44C8.1 | | | cyp-33C4 | | 0.008501 | 0.009456 | 0.00928 | | 0.003759 | | 0.000852 | | 0.000366 | | - |
| 4743 | F01D5.9 | | | cyp-37A1 | | - | - | 0.000454 | | - | | - | | - | | - |
| 5567 | F41B5.7 | | | cyp-33C6 | | - | - | - | | 0.007068 | | - | | - | | - |
| 6115 | C34B7.3 | | | cyp-36A1 | | - | - | - | | 0.004782 | | 5.06E-03 | | - | | - |
| 6689 | T10B9.3 | | | cyp-13A6 | | - | 8.00E-05 | - | | 0.000307 | | - | | 0.005053 | | - |
| 7239 | T10B9.8 | | | cyp-13A1 | | - | - | - | | 9.00E-06 | | 0.000137 | | 0.000995 | | - |
| 8681 | F14F7.2 | | | cyp-13A11 | | - | - | - | | - | | 0.000953 | | - | | - |
| 8997 | K09A11.3 | | | cyp-14A2 | | 0.003 | 0.008846 | 8.90E-05 | | - | | - | | - | | 0.000481 |
| 10005 | F08F3.7 | | | cyp-14A5 | | 0.006231 | 0.001064 | 0.00051 | | - | | - | | - | | 0.002497 |
| 11472 | T19B10.1 | | | cyp-29A2 | | - | 0.002399 | 1.00E-06 | | - | | - | | - | | 0.005803 |
| 11882 | Y5H2B.6 | | | cyp-33C12 | | - | - | - | | 0.000639 | | 0.000192 | | 0.001058 | | - |
| 11960 | Y80D3A.5 | | | cyp-42A1 | | - | - | - | | 0.000468 | | 3.10E-05 | | 0.001492 | | - |
| 12422 | C06B3.3 | | | cyp-35C1 | | - | - | 0.001448 | | - | | - | | - | | - |
| 12703 | H02I12.8 | | | cyp-31A2 | | - | - | - | | - | | 0.006139 | | - | | - |
| 12807 | B0213.10 | | | cyp-34A5 | | - | - | - | | 2.90E-05 | | 2.50E-05 | | 7.60E-05 | | - |
| 13326 | T09H2.1 | | | cyp-34A4 | | - | - | - | | 0.00109 | | 0.001786 | | 0.000444 | | - |
| 14001 | C36A4.1 | | | cyp-25A1 | | 0 | 0 | 0 | | - | | - | | - | | 5.00E-06 |
| 14472 | C49C8.4 | | | cyp-33E1 | | - | - | - | | 0.001268 | | 0.0088 | | 0.002975 | | - |
| 14784 | Y38C9B.1 | | | cyp-29A3 | | - | 0.003971 | 0.001313 | | - | | - | | - | | - |
| 15822 | K07C6.5 | | | cyp-35A5 | | - | 0.000381 | - | | - | | - | | - | | - |
| 16679 | B0213.12 | | | cyp-34A7 | | - | - | - | | 0.005865 | | 0.002263 | | 0.000708 | | - |
| 16861 | ZK1320.4 | | | cyp-13A10 | | - | - | - | | 0.000193 | | 0.006278 | | 0.00171 | | - |
| 17043 | F02C12.5 | | | cyp-13B1 | | - | - | - | | 0.002661 | | - | | 0.008914 | | - |
| 17078 | Y17G9B.3 | | | cyp-31A3 | | - | - | - | | 0.004409 | | 0.002877 | | - | | - |
| 20521 | C36A4.1 | | | cyp-25A1 | | - | 0.002156 | - | | - | | - | | - | | - |
| 20559 | C49G7.8 | | | cyp-35A4 | | - | - | 0.00316 | | - | | - | | - | | - |
| 21745 | C36A4.6 | | | cyp-25A4 | | - | - | - | | 0 | | 2.00E-06 | | 0.000561 | | - |

**Supplementary Table 7:** List of significantly regulated cytochrome P450 genes by CPF, DZN and temperature. Both tables show the microarray ID number. the sequence name, the gene name and the significant p-values (< 0.001) per treatment. For simplicity, p-values above the threshold had been removed. Supplementary table S11 show all the values as well as p-values for genes coding for SDR, UDP and GST coding genes. Table 3A includes all the significantly affected CYP by at least one of the treatments using a model that only considered no interacting effects. Table 3B includes all the affected CYP genes by at least one of the treatments using a model that considered interacting effects. For simplicity, p-values below the threshold have been removed.
