# Supplementary material for: Gene Expression Modifications by Temperature-Toxicants Interactions in Caenorhabditis elegans
Source: PLoS One. 2011 Sep 9;6(9):e24676. doi: 10.1371/journal.pone.0024676 (PMC3170376; doi:10.1371/journal.pone.0024676)
Supplement: Table S9 — Number of significantly expressed genes per variable in the full model ( Figure 5 ) and the overlap between variables. Numbers of gene per variable are indicated in bold (diagonal). Since a gene may be affected by more than one variable, the overlap between variables is also indicated. The last row shows the number of genes significantly affected by only one variable. (DOC) [file pone.0024676.s013.doc]

**Supplementary Table S9.-** Significantly expressed genes per variable**.** We analyzed the transcriptional effect of chlorpyrifos (CPF) and diazinon (DZN), at different temperatures (Temp). Genes which expression level was affected by those three environmental factors were identified with a linear model that considered also interaction between variables. In this way, genes significantly affected (-log p-value > 2) by CPF, DZN, Temp or any interaction between them (CPF*Temp, DZN*Temp, CPF*DZN*Temp) were identified. Numbers of gene per variable are indicated in bold (diagonal). Since a gene may be affected by more than one variable, the overlap between variables is also indicated. The last row shows the number of genes significantly affected by only one variable.

|  | **CPF** | **DZN** | **Temp** | **CPF*Temp** | **DZN* Temp** | **CPF*DZN** | **CPF*DZN*Temp** |
| --- | --- | --- | --- | --- | --- | --- | --- |
| **CPF** | **1341** |  |  |  |  |  |  |
| **DZN** | 821 | **1248** |  |  |  |  |  |
| **Temp** | 1180 | 997 | **1932** |  |  |  |  |
| **CPF*temp** | 249 | 256 | 319 | **2930** |  |  |  |
| **DZN*temp** | 185 | 174 | 233 | 1570 | **2230** |  |  |
| **CPF*DZN** | 252 | 265 | 327 | 1970 | 1710 | **2995** |  |
| **CPF*DZN*temp** | 733 | 715 | 809 | 182 | 130 | 189 | **1061** |
| **Unique** | 69 | 96 | 371 | 655 | 256 | 574 | 98 |
